# Tumour homing and therapeutic effect of colloidal nanoparticles depend on the number of attached antibodies

**DOI:** 10.1038/ncomms13818

**Published:** 2016-12-19

**Authors:** Miriam Colombo, Luisa Fiandra, Giulia Alessio, Serena Mazzucchelli, Manuela Nebuloni, Clara De Palma, Karsten Kantner, Beatriz Pelaz, Rany Rotem, Fabio Corsi, Wolfgang J. Parak, Davide Prosperi

**Affiliations:** 1 Dipartimento di Biotecnologie e Bioscienze, Università di Milano-Bicocca, Piazza della Scienza 2, 20126 Milano, Italy; 2 Ospedale L. Sacco, Via G. B. Grassi 74, 20157 Milano, Italy; 3 Dipartimento di Scienze Biomediche e Cliniche ‘Luigi Sacco', Università di Milano, Via G. B. Grassi 74, 20157 Milano, Italy; 4 Fachbereich Physik, Philipps Universität Marburg, Renthof 7, 35037 Marburg, Germany; 5 Surgery Department, Breast Unit, ICS Maugeri S.p.A. SB, Via S. Maugeri, 10-27100 Pavia, Italy; 6 CIC Biomagune, Miramon Pasealekua 182, 20009 San Sebastian, Spain

## Abstract

Active targeting of nanoparticles to tumours can be achieved by conjugation with specific antibodies. Specific active targeting of the HER2 receptor is demonstrated *in vitro* and *in vivo* with a subcutaneous MCF-7 breast cancer mouse model with trastuzumab-functionalized gold nanoparticles. The number of attached antibodies per nanoparticle was precisely controlled in a way that each nanoparticle was conjugated with either exactly one or exactly two antibodies. As expected, *in vitro* we found a moderate increase in targeting efficiency of nanoparticles with two instead of just one antibody attached per nanoparticle. However, the *in vivo* data demonstrate that best effect is obtained for nanoparticles with only exactly one antibody. There is indication that this is based on a size-related effect. These results highlight the importance of precisely controlling the ligand density on the nanoparticle surface for optimizing active targeting, and that less antibodies can exhibit more effect.

## Abstract (web summary)

A common strategy to target nanoparticles to tumours is conjugation with specific antibodies, targeting protein expressed preferentially by cancer cells. Here the authors show that the number of antibodies bound to the nanoparticle influences the targeting ability *in vitro* and *in vivo*.

In the last 10 years, the exponential growth of nanotechnology has led to new opportunities in biology and medicine, from the development of highly sensitive biosensors for the detection of molecular interactions to novel medical diagnostic tools, from therapeutic applications in oncology to drug delivery systems, and from cellular therapy to tissue engineering^1^,^2^,^3^. Colloidal nanoparticles (NPs), including metal, magnetic and semiconductor NPs, are a versatile tool to integrate nanotechnology and biology, provided that they are complemented with a proper surface functionalization. To this aim, several strategies have been proposed in the attempt to optimize the surface modification of NPs with organic and biological targeting ligands to improve the NP affinity towards biological receptors^4^. However, recent developments have pointed out that a few requirements should be taken into account in designing bio-targeted NPs, including tight irreversible (or reversible) binding, and control on density and orientation^5^,^6^,^7^,^8^. While several studies have been carried out to achieve a control on linkage stability^9^, as on ligand orientation and density^10^, besides some examples^11^,^12^, a general strategy to introduce a discrete precisely controlled number of targeting biomolecules to each NP is still largely missing. Importantly, this makes it difficult to provide direct evidence on the relationship between the extent of NP functionalization and the targeting efficiency of the NP as a selective diagnostic tool or a drug delivery system. In this work, we propose a straightforward method to introduce a discrete number of biomolecules (here in the form of antibodies) on NPs for biomedical applications. We developed a nanostructured probe consisting of colloidal polymer-coated Au NPs functionalized on their surface with a defined discrete number of trastuzumab (Tz) molecules. This monoclonal antibody recognizes the HER2 receptor (human epidermal growth factor receptor 2), which is overexpressed in about 25–30% of breast tumours, and its clinical use has significantly changed the natural history of HER2-positive breast cancer (BC). By recognizing the HER2 receptor, Tz blocks its downstream signalling activity^13^ and interferes with the PI3K survival pathway^14^, therefore causing cell cycle arrest and inhibition of cancer proliferation. Moreover, Tz is able to activate the antibody-dependent cellular cytotoxicity by recruitment of natural killer cells^15^. Therefore, Tz nanoconjugates are expected to combine a focused targeting action with a therapeutic effect on HER2-positive tumours. In this work we report a method to synthesize NPs with a precisely controlled number of attached antibodies, that is, exactly one or exactly two Tz antibodies per NP, with the goal of probing the dependence of the antibody density on the NP surface on *in vitro* and *in vivo* targeting efficiency. Unexpectedly, we find that despite an intuitive belief that *in vivo* targeting efficiency should rise on increasing the amount of antibodies per NP, the tumour homing and protracted therapeutic efficacy are best achieved with just one antibody attached per NP. As NPs with nonspecific IgG antibodies were used as control, our data also allowed to distinguish between active, that is, Tz-mediated binding, versus passive targeting, that is, tumour enrichment of NPs due to enhanced permeation and retention (EPR) effect. Data show that only in case of one Tz antibody per NP, but not in case of two Tz antibodies per NP, the effect of active targeting could be observed.

## Results

### NP preparation and characterization

**Figure 1:**
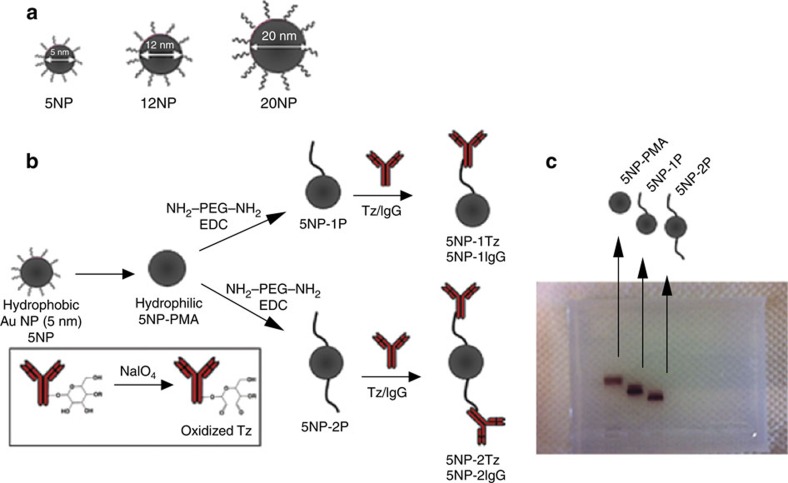
Synthesis and conjugation of NPs. (**a**) Surfactant-coated Au NPs of 5 (5NP), 12 (12NP) and 20 (20NP) nm. (**b**) Synthesis scheme. Hydrophobic Au NPs are transferred from organic solvent to aqueous solution by overcoating them with an amphiphilic polymer. The surface of the polymer-coated NPs is conjugated with diamine–PEG using EDC chemistry. Using gel electrophoresis, conjugates with exactly one (5NP-1P) or two (5NP-2P) PEG molecules attached per NP are obtained. Tz antibody is oxidized with NaIO_4_ and linked to the free terminals of PEG, leading to NPs with one (5NP-1Tz) or two (5NP-2Tz) antibodies per NP. (**c**) Image of an agarose gel with NP, NP-1P and NP-2P.

The 5 nm core diameter Au NPs were synthesized according to the Brust–Schiffrin protocol^16^ in organic phase and transferred to aqueous solution by overcoating them with an amphiphilic polymer^17^. For purpose of visualization, organic dyes (fluorescein isothiocyanate (FITC) or Alexa Fluor 660 (AF660)) were optionally integrated into the polymer shell^18^. The resulting NPs had a core diameter of *d*_c_=4.8±1.3 nm (s.d.; Supplementary Fig. 1), a hydrodynamic diameter of *d*_h_=8.28±0.63 nm and a zeta potential of *ζ*=–23.7±1.8 mV in water (Supplementary Table 1). Colloidal properties, toxicity and biodistribution of these NPs have been characterized in detail and we refer to previous publications^19^,^20^ (Supplementary Figs 1 and 2). Polyethylene glycol (*bis*-amino-PEG, *M*_w_≈10 kDa) was linked covalently to the surface of the NPs by standard bioconjugate chemistry and the resulting NP-PEG conjugates were fractionated by agarose gel electrophoresis. This allowed us to extract NPs with exactly one or exactly two PEG molecules attached per NP (5NP-1P and 5NP-2P, respectively)^21^. For linkage of Tz antibody to the terminal amino group of PEG, the carbohydrate unit of the vicinal rings in the Fc region of the Tz antibodies was mildly oxidized with sodium periodate. This led to reactive aldehyde groups, resulting in an imine bond, which was *in situ* reduced giving a stable secondary amine bridge^22^. As the saccharide moiety is placed in the Fc portion of the antibody, this conjugation strategy was well suited for our study because (1) all the Tz molecules were positioned with the same arrangement on the NP surface and (2) the Fab binding site was presented in an optimal orientation for receptor binding on each NP (Fig. 1).

**Figure 2:**
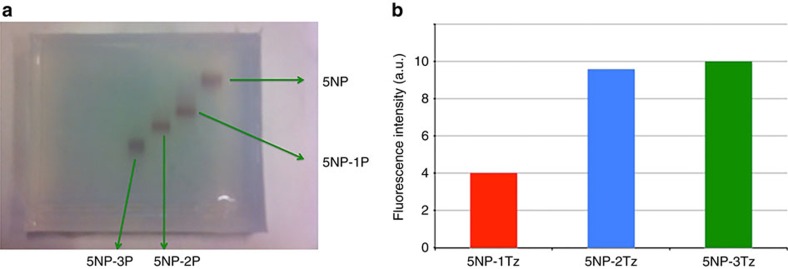
Functionalisation of 5 nm Au NPs with a discrete number of ligands. (**a**) Image of an agarose gel with 5NP, 5NP-1P, 5NP-2P and 5NP-3P conjugates. (**b**) Fluorescence intensity of 5NP-1Tz, 5NP-2Tz and 5NP-3Tz, whereby each Tz molecule is labelled with one AF660 fluorophore.

Conjugation was verified by observing the increase in hydrodynamic diameters with dynamic light scattering (DLS), as by dot blot analysis (Supplementary Table 1 and Supplementary Figs 3 and 4). The difference in ligand density (one versus two antibodies per NP) was investigated by quantification of protein corona formation around the NPs (without fluorescence label in their polymer shell) by incubating them with FITC-labelled bovine serum albumin (BSA). As expected, the surface functionalization with Tz decreased the extent of protein adsorption (as quantified by measurements of FITC fluorescence intensity (FI)), which in the case of NPs with only one Tz antibody attached per NP (that is, 5NP-1Tz), was twofold higher than in the case of two Tz antibodies attached per NP (that is, 5NP-2Tz). This result confirmed a double-accessible surface area due to the different extent of functionalization (Fig. 2 and cf. the Methods section for experimental details and Supplementary Figs 5 and 6). The stability of the NPs in serum was evaluated up to 96 h by DLS analysis. We found that both kinds of NPs maintained the original hydrodynamic size (Supplementary Table 2). Besides the direct detection of double fluorescence emission in NPs with two attached Tz (5NP-2Tz) as compared with NPs with one Tz attached (5NP-1Tz; Fig. 1b), we attempted to achieve confirmation on the final number of antibody molecules per NP. Unfortunately, both Fourier transform infrared spectroscopy analysis and transmission electron microscopy (TEM)-negative staining did not provide conclusive results due to strong interference from the organic polymer coating. However, robust previous evidence with different molecular structures or even larger objects such as multiple NPs corroborate our conclusion^21^,^23^. Fluorescence of the NPs with attached Tz was not affected by incubation in human plasma (Supplementary Tables 3 and 4).

### *In vitro* targeting and cell viability

**Figure 3:**
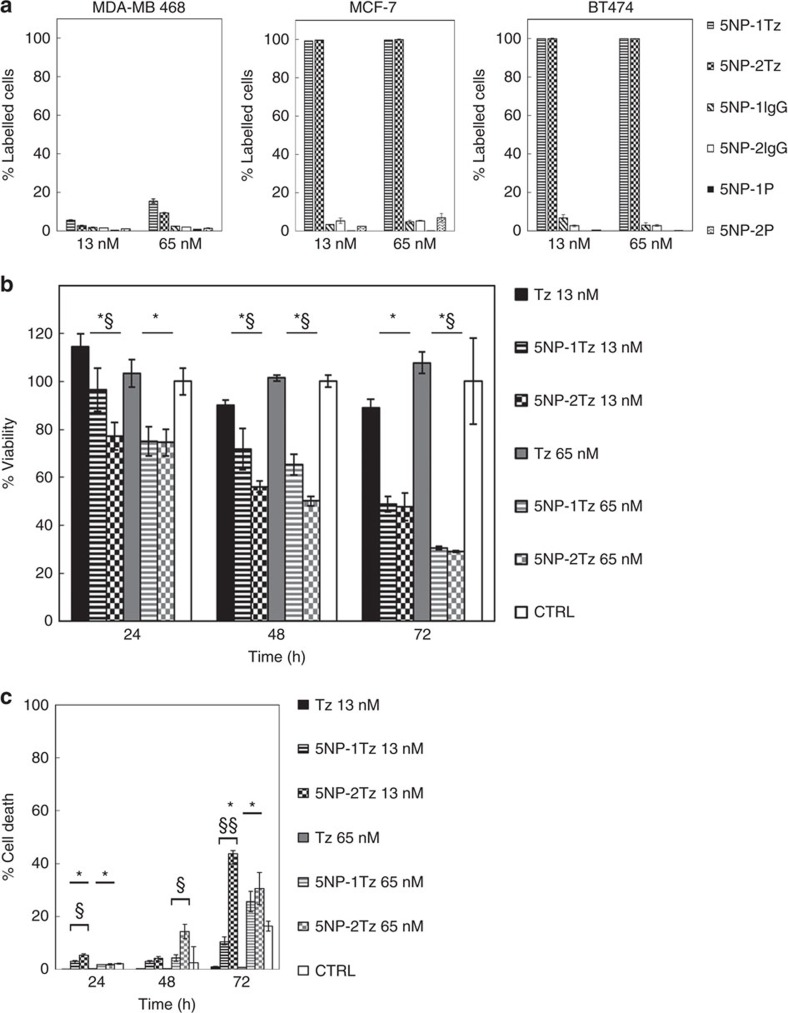
*In vitro* assays. (**a**) Binding assay with BC cells. MCF-7, BT474 and MDA-MB 468 cells were incubated at 37 °C for 1 h with 13 and 65 nM FITC-labelled NPs functionalized with 5NP-1Tz and 5NP-2Tz, and then processed with flow cytometry, in which labelling of cells with NPs was detected. FITC-labelled NPs functionalized with generic rabbit IgG (IgG) or only with PEG were used as specificity controls. Untreated cells were used to create viable cells and singlet gates, and to set the positive region assigned to labelled cells. (**b**) Viability of cells treated with NPs. MCF-7 cells were treated with 13 and 65 nM of NPs or Tz for up to 72 h. Viability was tested by measuring the conversion of MTT into formazan. Reported values are the mean of six replicates±s.e., normalized on cell proliferation of untreated MCF-7 cells (control), respectively. *P<0.01, 5NP-1Tz or 5NP-2Tz versus control; ^§^*P*<0.01, 5NP-1Tz versus 5NP-2Tz antibodies (Student's *t*-test). (**c**) Cell death assay on incubation with NPs. MCF-7 cells were treated with 13 and 65 nM of NPs for 24, 48 and 72 h. Untreated cells and Tz-treated cells (13 and 65 nM) were used as control. Cell death was assessed based on the exposure of Annexin V, evaluated by flow cytometry. Live cells were used to set the region of positivity. Reported values are the mean of three replicates±s.e. *P<0.01, 5NP-1Tz or 5NP-2Tz versus control; ^§^*P*<0.01; ^§§^*P*<0.0005, 5NP-1Tz versus 5NP-2Tz (Student's *t*-test). CTRL, control.

The targeting efficiency and selectivity of 5NP-1Tz and 5NP-2Tz towards HER2 were first assessed using MDA-MB 468, MCF-7 and BT474 cell lines, which showed basal, moderate or high expression of HER2, respectively (Fig. 3 and Supplementary Fig. 7). Cells were treated with different concentrations of NP-Tz conjugates with FITC label in the polymer shell of the NPs (13 and 65 nM NP concentration) at 37 °C for 1 h, to simulate *in vitro* physiological interaction between cells and NPs. The amount of NP-Tz conjugates attached to cells was then assessed by flow cytometry (Fig. 3a). NPs conjugated to one or two generic anti-rabbit IgG molecules, or only to one or two PEG molecules per NP, were used as negative controls, while untreated cells were used to set the singlet gate and the positive region in which cells were identified as decorated with NPs. In MCF-7 and BT474, the percentage of cells in the positive region in the case of NP-Tz conjugates was at least 10-fold higher than the one in the controls, demonstrating the specificity of the NP-cell targeting via Tz-HER2 recognition. Moreover, the percentage of cell labelling in these cell lines was almost 100% even at the lowest concentration, suggesting that 13 nM NP-Tz conjugates had already saturated the signal. The NP-Tz conjugates displayed a very low and dose-dependent percentage of labelled MDA-MB 468 cells, in accordance to the poor HER2 expression. The recognition mediated by Tz maintained its specificity, as suggested by statistical significance in comparison with controls (*P*<0.05, Student's *t*-test). In addition, the analysis performed with MDA-MB 468 cells pointed out that the immobilization of two Tz molecules per NP did not seem to confer any advantage in cell recognition *in vitro*. Indeed, excluding aggregated cells bridged by individual 5NP-2Tz by means of singlet gate data analysis we did not observe any increase in the percentage of labelled cells in samples treated with 5NP-2Tz in comparison with 5NP-1Tz. However, these data could not exclude that a NP with two Tz antibodies attached is instead capable of bridging two HER2 receptors on the same cell, resulting in a more pronounced therapeutic effect as suggested by viability and cell death assays (Fig. 3b,c).

MDA-MB 468 and BT474 cells were excluded from next experiments due to their too low and too high HER2 expression, respectively. MCF-7 cells were instead selected to develop the *in vivo* model because their moderate HER2 expression could allow us to better appreciate differences in tumour recognition and accumulation of 5NP-1Tz versus 5NP-2Tz. The decrease in MCF-7 cell viability due to the treatment with NPs was statistically significant at all concentrations and times tested, and was dose- and time-dependent (Fig. 3b). We also observed differences between samples treated with 5NP-1Tz or 5NP-2Tz, which suggests a correlation between the amount of antibody immobilized on the NP and the toxicity of the nanoconjugate. However, since both 13 and 65 nM of free Tz were not effective under the tested conditions, our findings suggest a synergistic contribution between NPs and Tz to cellular toxicity. Cell death induced by NPs in comparison with untreated cells was statistically significant after 72 h of incubation. As expected, a better antibody-mediated antitumour activity of NPs with two antibodies per NP was observed. This is probably due to the availability of a double amount of antibody molecules, although free Tz does not seem to be effective under the same conditions (Fig. 3c). However, any possible contribution to significant cytotoxic effects derived from Au NPs without ligands attached was ruled out by previous data obtained at the same concentrations used in this work^19^.

**Figure 4:**
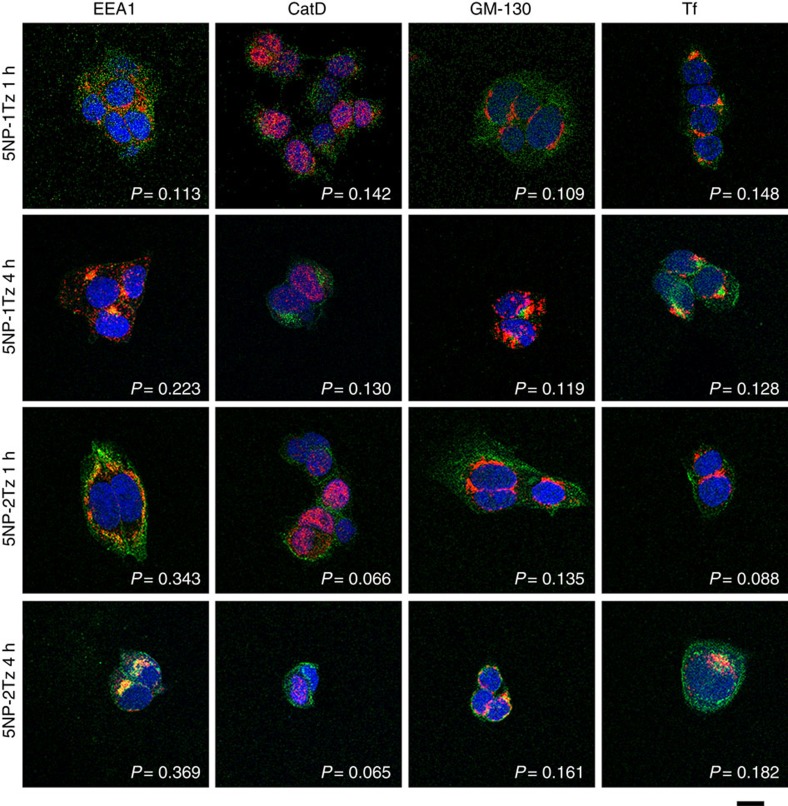
Intracellular localization of 5NP-1Tz and 5NP-2Tz. Confocal microscopy images of MCF-7 cells, incubated for 1 or 4 h at 37 °C with 65 nM of %NP-1Tz or 5NP-2Tz (FITC fluorescence in green). Early endosomes, lysosomes, Golgi and recycling endosomes were recognized, respectively, with the early endosome marker EEA1, the lysosomal protein CatD, the Golgi marker GM130 and the recycling endosome marker Tf antibodies, and labelled with an anti-mouse secondary antibody conjugated with Alexa Fluor 546 (red; Invitrogen). Nuclei were stained with DAPI (blue). The scale bar corresponds to 10 μm. Pearson's correlation coefficients (*P*) have been calculated using the image J plugin JaCoP.

The cellular uptake of 5NP-1Tz and 5NP-2Tz was studied by confocal microscopy (Fig. 4). Images are consistent with an early-stage partial compartmentalization of 5NP-1Tz in endosomes only after 4 h, while 5NP-2Tz exhibited detectable co-localization already after 1 h. This result suggested that 5NP-2Tz are internalized in HER2^+^ cells faster than 5NP-1Tz. Within this time intervals, no co-localization with other organelles was observed in both cases.

### *In vivo* targeting and biodistribution

**Figure 5:**
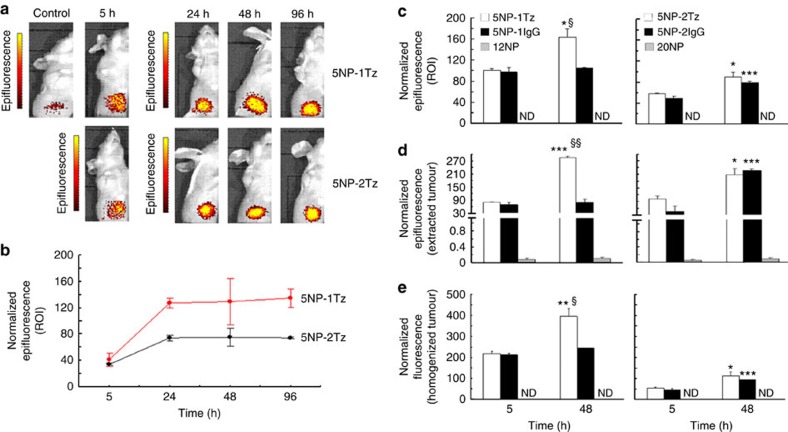
*In vivo* tumour targeting. Epf images of (**a**) mice bearing MCF-7 xenografts and (**b**) averaged Epf intensity of the tumour ROI are shown, acquired 5, 24, 48 or 96 h after exposure to 5NP-1Tz and 5NP-2Tz labelled with AF660. The colour scales in **a** indicate fluorescence expressed as radiant efficiency ((p s^−1^ cm^−2^ sr^−1^) (μW cm^−2^)^−1^), where p s^−1^ cm^−2^ sr^−1^ is the number of photons per second that leave a square centimetre of tissue and radiate into a solid angle of one steradian (sr). The scale extends from the following minimum–maximum values (left to right, top to bottom): 4.94 × 10^7^–6.03 × 10^7^; 1.19 × 10^8^–1.50 × 10^8^; 8.45 × 10^7^–1.01 × 10^8^; and 1.30 × 10^8^–1.80 × 10^8^. In the control mouse no injection was performed. Averaged Epf intensity of (**c**) tumour ROI and (**d**) isolated tumours are shown, acquired 5 or 48 h after exposure to non-functionalized 12 (12NP) or 20 nm (20NP) NPs, or to 5NP-1Tz and 5NP-2Tz or 5NP-1IgG and 5NP-2IgG, labelled with AF660. In **e**, fluorescence intensities (FIs) of tumour homogenates are displayed. Epf and FI values were normalized to the FI of injected solution to keep into account the differences in intrinsic fluorescence emission for each NP-antibody conjugate. Mean value±s.e. of three different samples for each experimental condition are provided. *P<0.05, **P<0.01 and ***P<0.001 versus 5 h; ^§^*P*<0.01 and ^§§^*P*<0.001 versus 5NP-1Tz (Student's *t*-test). ND, non-detectable.

**Figure 6:**
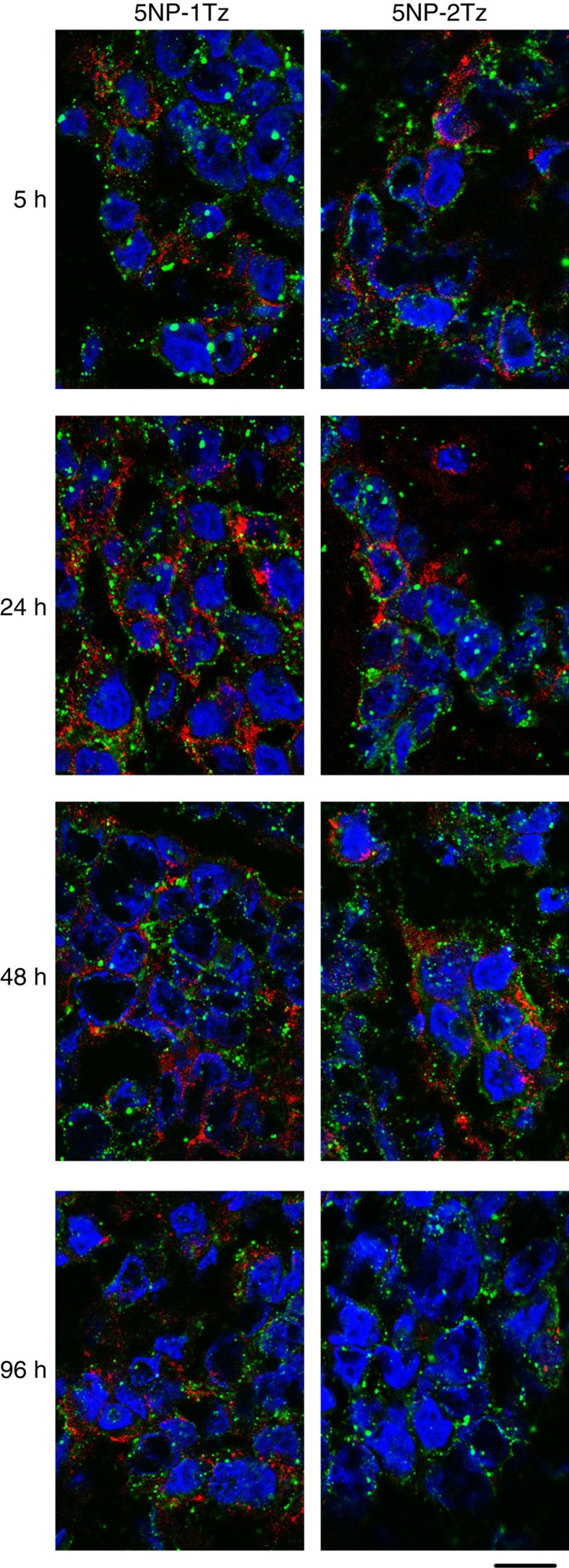
Fluorescence confocal microscopy of tumours. Confocal laser scanning micrographs (single optical sections) of cryosections obtained from MCF-7 tumours isolated 5, 24, 48 or 96 h after exposure to 5NP-1Tz or 5NP-2Tz, labelled with AF660, and then counterstained with anti-cytokeratin19 and DAPI for tumour cells and nuclei detection, respectively. The confocal images of NPs (red) have been overlaid on the corresponding images reporting nuclei (blue) and cells (green). The scale bar corresponds to 10 μm.

**Figure 7:**
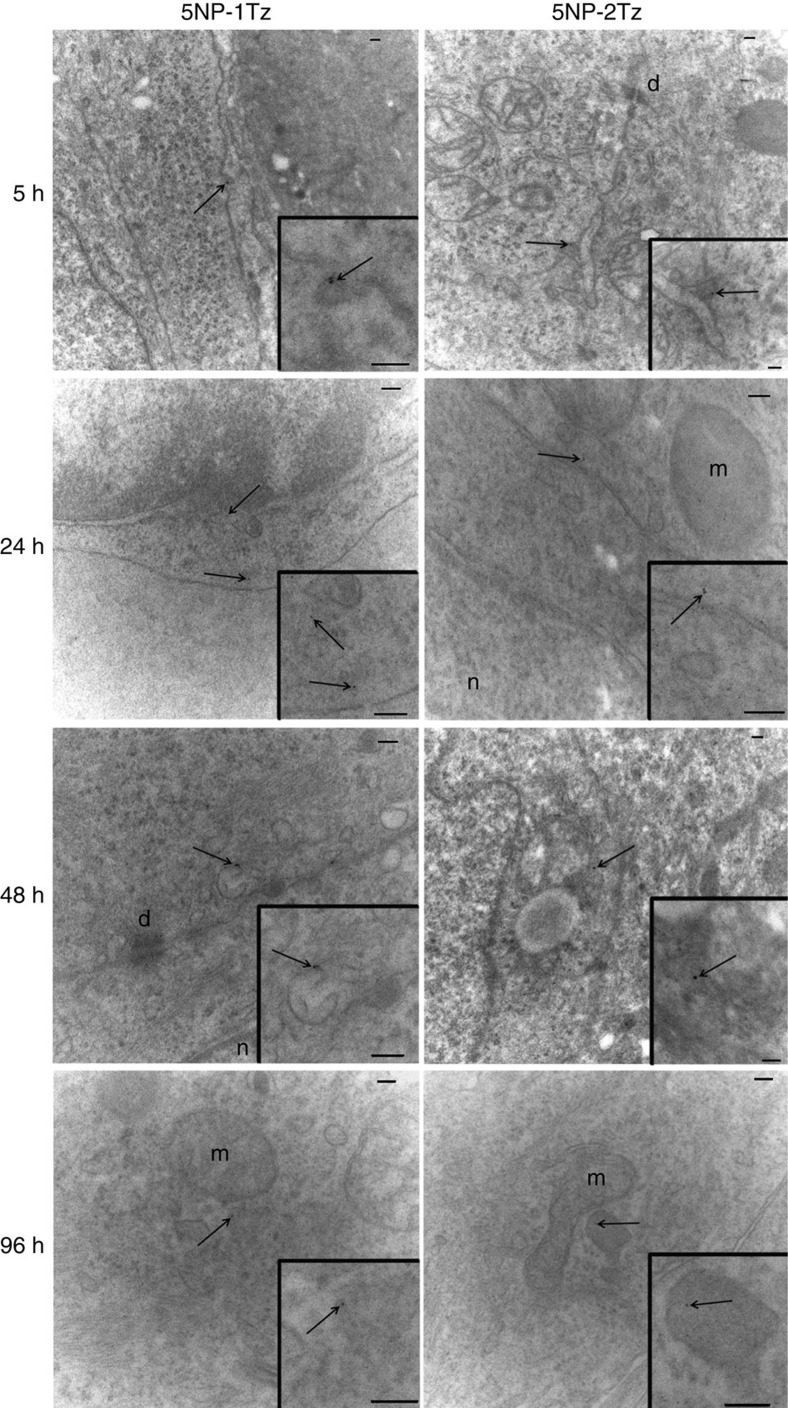
Transmission electron microscopy of tumours. TEM images of MCF-7 tumours isolated 5, 24, 48 or 96 h after exposure to 5NP-1Tz or 5NP-2Tz. In the insert of each single figure, the corresponding higher magnification image is reported. NPs are evidenced by arrows; d, desmosome; n, nucleus; m, mitochondrion. The scale bars correspond to 100 nm.

Two groups of Balb/c nude mice, bearing a subcutaneous MCF-7 BC, were treated with NP-Tz conjugates (18 pmol g^−1^ body weight). Labelling of antibodies with AF660 dye proved to be more reliable than direct NP labelling and was then preferred. NP-Tz conjugates were injected in mice by the tail vein and their availability in plasma and localization at the tumour was monitored 5, 24, 48 and 96 h after injection. The quantification of gold in plasma, obtained by inductively coupled plasma mass spectrometry (ICP-MS), revealed that the blood half-life calculated for the monofunctionalized NPs was not different from that of the bifunctionalized NPs (Supplementary Table 5), and therefore the biodistribution and the tumour homing of the nanoformulations was not affected by their availability in plasma between 5 and 96 h after injection. To detect the localization of the NPs into the xenografts, epifluorescence (Epf) images of anaesthetized supine mice were obtained by an IVIS Lumina II imaging system. Figure 5a indicated that the NP-Tz conjugates were able to target the HER2^+^ tumours at 5 h post injection. A strong increase in tumour Epf was observed after 24 h, which did not vary over the following 48 h. 5NP-2Tz showed twofold higher intrinsic FI compared with 5NP-1Tz, due to the double number of labelled antibodies on each NP. Therefore, the Epf average values of the region of interest (ROI) on the Epf intensity of the two solutions was normalized. Four mice per experimental condition were analysed. Our results showed a twofold higher Epf normalized intensity at the tumour when 5NP-1Tz were injected compared with mice treated with 5NP-2Tz, surprisingly suggesting a stronger propensity of conjugates with one versus two antibodies attached to each NP to accumulate at HER2^+^ tumour (Fig. 5b). Confocal microscopy analysis of tumour cryosections isolated between 5 and 96 h post injection showed that both types of NP-Tz conjugates came into contact with the BC cells to be then internalized (Fig. 6). However, while fluorescence of 5NP-2Tz completely decayed at 96 h post injection, the signal from 5NP-1Tz was still detectable in BC cells. TEM images of the same samples (Fig. 7) provided evidence that both types of NP-Tz conjugates were captured by the plasma membrane of BC cells within the first 5 h, and membrane invagination indicated binding of the NPs to the target receptors activating the process of internalization. As expected, both types of conjugates were then directed to endosomes (24 and 48 h). However, only 5NP-2Tz were found in lysosomes at 96 h post injection, where the degradation of the fluorescent protein material likely occurs (Fig. 7), while 5NP-1Tz were still observed in endosomes.

The systemic biodistribution of the injected NP-Tz conjugates was also investigated by analysing the Epf of mice bladder ROI at 5, 24, 48 and 96 h post injection. Supplementary Fig. 8 shows that (at least the fluorescence polymer part of) both types of conjugates were eliminated by kidney excretion, with a higher extent for 5NP-1Tz as compared with 5NP-2Tz, which is likely due to the smaller size^8^,^20^. The maximal amount of NPs (or more accurately of the fluorescence polymer part of the NPs) inside the bladder was recorded at 24 h post injection, and decreased over the following hours. ICP-MS was used to quantify the amount of gold from the NP-Tz conjugates in tumour, heart, liver, lungs, kidneys and spleen dissected at different experimental times (Supplementary Fig. 9). The results confirmed a higher accumulation of the monofunctionalized NPs in tumours as compared with the bifunctionalized ones (Fig. 5). Moreover, only 5NP-1Tz exhibited a progressive increase in gold content over time, while the amount of 5NP-2Tz in the tumour reached a maximum at 24 h post injection and decreased over the following hours, to remain almost undetectable at 96 h. Liver and spleen were the two off-target organs mainly subjected to gold localization, while the NP-Tz conjugates did not accumulate in kidneys, where the NPs likely underwent filtration to be rapidly excreted via the bladder. Unlike tumours, the gold content of 5NP-1Tz in liver and spleen decreased after 24 h, while an increasing trend over time was observed for 5NP-2Tz up to 96 h. Hence, 5NP-1Tz as compared with 5NP-2Tz seem to be preferable for tumour targeting *in vivo*, as they accumulate in a larger amount at the malignant site where they are subjected to a slower degrading process. Moreover, monofunctionalized NPs undergo a more efficient renal clearance compared with the bifunctionalized ones, with a reduced accumulation in off-target organs at longer times of exposure.

**Figure 8:**
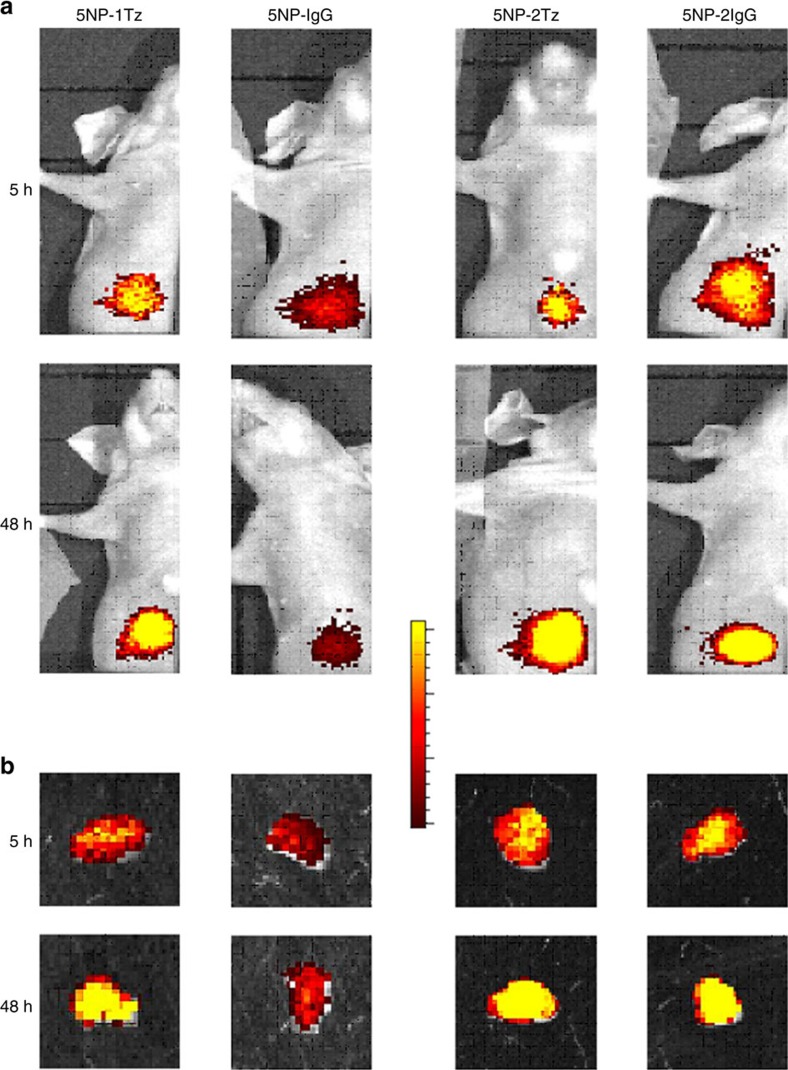
*In vivo* active targeting contribution. (**a**) Epf images of mice bearing MCF-7 xenografts and (**b**) isolated tumours, acquired 5 or 48 h after exposure to 5NP-1Tz, 5NP-1IgG, 5NP-2Tz or 5NP-2IgG, labelled with AF660. The minima and maxima of the logarithmic colour scales are in (p s^−1^ cm^−2^ sr^−1^) (μW cm^−2^)^−1^ (from left to right): (**a**) 5 h: 3.46 × 10^7^–6.06·10^7^; 3.46 × 10^7^–6.06 × 10^7^; 5.79 × 10^7^–7.04 × 10^7^; and 5.79 × 10^7^–7.04·10^7^; (**a**) 48 h: 7.91 × 10^7^–1.00 × 10^8^; 3.46 × 10^7^–6.06 × 10^7^; 8.43 × 10^7^–1.11 × 10^8^; and 8.43 × 10^7^–1.11 × 10^8^; (**b**) 5NP-1Tz and 5NP-1IgG: 1.85 × 10^6^–8.23 × 10^6^; (**b**) 5NP-2Tz and 5NP-2IgG: 3.79 × 10^6^–1.43 × 10^7^.

The importance of active molecular recognition in targeting MCF-7 cells *in vivo* was assessed. In detail, the accumulation of the two types of HER2-targeted NP-Tz conjugates in the tumour mass of mice was compared with tumour distribution of the same NPs functionalized with a nonspecific rabbit IgG antibody. From the *in vivo* and *ex vivo* fluorescence images of the tumours exposed for 5 and 48 h to the targeted and non-targeted NPs (Fig. 8), we derived Epf values that were normalized to the Epf intensity of the different solutions. Normalization was achieved by dividing the fluorescence emission of an injectable solution of 5NP-2Tz at the same concentration of a solution of 5NP-1Tz by a factor of 2. Figure 5c,d confirmed the more efficient tumour targeting of 5NP-1Tz as compared with 5NP-2Tz *in vivo*, suggesting a greater access of the monofunctionalized NPs to BC cells. This is likely to be a size-related issue, as in fact, the tumour Epf values of the mice treated with IgG-NP conjugates with one IgG antibody per NP (5NP-1IgG), indicative of the passive, that is, EPR contribution, were also higher than those recorded in the mice exposed to conjugates with two IgG antibodies per NP (5NP-2IgG) (Fig. 5c,d). As expected, the Epf intensity of the tumours exposed to HER2-targeted NPs significantly increased after 48 h. Nevertheless, while no significant change in the Epf intensity was observed for 5NP-1IgG, an enhanced Epf signal was recorded for 5NP-2IgG, comparable to that observed in mice treated with conjugate with two Tz per NP (Fig. 5c,d). The FI values of tumour homogenates, normalized on the FI of the injected solution (Fig. 5e), highlighted the higher accumulation in BC cells of 5NP-1IgG versus 5NP-2IgG, as a result of a higher EPR effect. Also, the difference in the tumour homing between both IgG-NP conjugates after 48 h post injection was confirmed. Therefore, the monofunctionalization of Au NPs was the only way to discriminate between the active and the passive tumour targeting at 48 h post injection. This was possible because the EPR effect in the mice treated with monofunctionalized NPs did not increase over time, as rather occurred in the mice exposed to bifunctionalized NPs. The exclusive role of the EPR effect on affecting the tumour targeting of bi- versus monofunctionalized NPs *in vivo* was confirmed by the confocal analysis of tumour cryosections (Supplementary Fig. 10), where no differences in the localization of the two types of HER2-targeted NPs were observed. This could be explained in terms of a smaller number of bifunctionalized NPs homing at the tumour compared with mono-, although those NPs that can reach the tumour tissue effectively bind to the HER-2 in MCF-7 cells. As expected, no interaction with the cells occurred in the presence of nonspecific NPs functionalized with one or two IgGs per NP (Supplementary Fig. 10).

To establish whether the size of the NPs, besides the different number of ligands, could affect tumour homing, we treated MCF-7 tumour-bearing mice with AF660-labelled non-functionalized Au NPs of 12 (12NP) or 20 nm (20NP) core diameter, resembling the size of mono- and bifunctionalized NPs, respectively (Supplementary Figs 11 and 12). We first measured the plasma half-life of these NPs by ICP-MS, resulting in values significantly lower than those of the HER2-targeted NPs (Supplementary Table 4). This result was in accordance with the surface charge of these non-PEGylated and non-targeted NPs, which was expected to promote the formation of the protein corona and the interactions with the immune system components. The localization of 12NP and 20NP into the tumour xenografts was then performed *in vivo* and *ex vivo*. We found that the tumour homing of these NPs at 5 and 48 h post injection was strongly affected by their low plasma availability, as demonstrated by the total lack of a detectable Epf signal in supine mice (Fig. 5c and Supplementary Fig. 13) and FI in tumour homogenates (Fig. 5e), and by a very low Epf in dissected tumours (Fig. 5d and Supplementary Fig. 13). The Epf of the tumours exposed to 12NP was comparable to that observed with 20NP between 5 and 96 h post injection (Supplementary Fig. 14). Therefore, we concluded that the higher efficiency of NPs with one versus two antibodies attached in accumulating in HER2^+^ tumours (Fig. 5a,b) is not caused simply by the reduced size of the NPs, but rather the number of surface ligands plays a crucial role. While the NP stability experiments, which are shown in Supplementary Table 2, demonstrate that there is no apparent desorption *in vitro*, complete integrity *in vivo* cannot be warranted. However, we could build on previous evidence that the polymer coating remains predominantly anchored to the NP core in plasma, although it is partly degradable in the liver^20^. In addition, due to the control 12NP and 20NP, and due to the control NPs with IgG, we can conclude that the targeting effect is specific for Tz-bearing NPs. The role of the PEG, which has been used as linker, has not been investigated in this study. While there may be some loss in targeting efficiency due to partial degradation, the effect of the remaining intact NPs is still dominating.

### Regulation of HER2 expression

**Figure 9:**
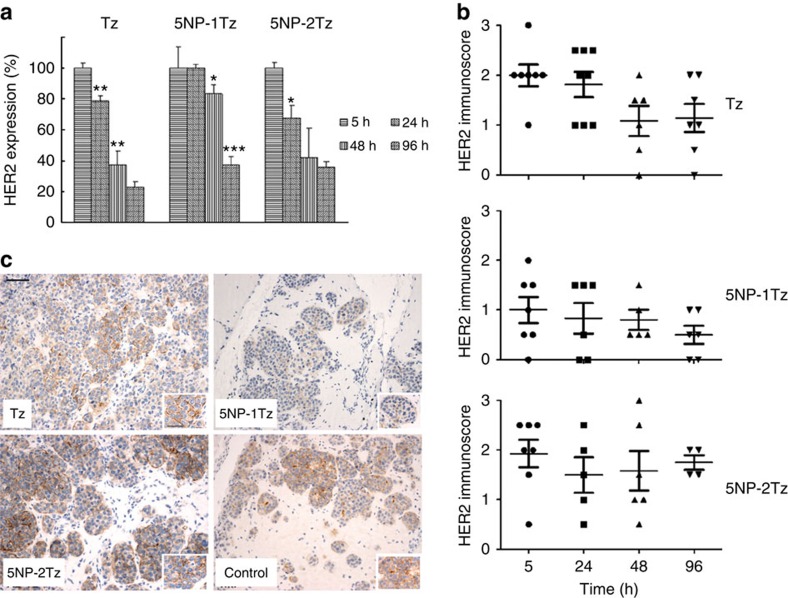
HER2 expression modulation. (**a**) Percentage variation of total HER2 expression in tumour homogenates and (**b**) immunoscore (from 0 to 3+) of HER2 transmembrane expression on tumour sections obtained from mice exposed to free Tz or 5NP-1Tz and 5NP-2Tz for 5, 24, 48 or 96 h post injection. *P<0.05, **P<0.01 and ***P<0.001 calculated comparing two consecutive time points. (**c**) HER2 expression on tumour tissues at 96 h post injection. The control corresponds to non-injected animals. Injection of Tz and of 5NP-2Tz showed intense and complete HER2 immunostaining on cellular membrane (brown colour), while in case of injection of 5NP-1Tz only rare cells were HER2^+^, with weak and incomplete cellular membrane staining ( × 20). High-magnification images of immunostaining are reported in insets ( × 40). The scale bars in the upper left picture in **c** correspond to 100 or 10 μm in low- or high-magnification images, respectively, and apply for all other images.

To get insights into the therapeutic effect of the as-characterized mono- and bifunctionalized Au NPs on BC, we investigated their regulatory activity onto the expression of HER2, the therapeutic target of Tz. Tumour-bearing mice were injected with NP-Tz conjugates (calculated to have 18 pmol per g body weight of Tz, both with 5NP-1Tz and 5NP-2Tz) or with a comparable amount of free Tz (18 pmol per g body weight), labelled with the AF660 dye. We first checked that injected Tz was indeed able to target BC in the MCF-7-based murine model, by assessing its accumulation at the tumour site by measuring the fluorescence from AF660 (Supplementary Fig. 15). Next, mice treated with 5NP-1Tz and 5NP-2Tz, respectively, were killed and dissected at 5, 24, 48 and 96 h post injection. Total HER2 expression in the tumour tissue homogenates from all mice was analysed by western blotting (Supplementary Figs 16 and 17) and the percentage variation of HER2 expression over time was calculated for each group of mice. HER2 expression recorded at 5 h post injection was considered the baseline in each group of treatment and was normalized to 100%. As shown in Fig. 9a, treatment of mice with Tz induced a progressive decrease of HER2 protein expression at 24 and 48 h, while at 96 h the decrease was no longer significant when compared with that recorded at 48 h. Treatment with 5NP-2Tz led to a significant reduction of HER2 protein only at 24 h, while a tendency towards decrease was suggested at 48 h; no additional decrease occurred at 96 h. Finally, the results obtained on treatment with 5NP-1Tz indicated that no variation in the total HER2 expression occurred within the first 24 h, while a pronounced decrease was detectable at 48 h with a further obvious decrease at 96 h post injection, thus indicating a sustained therapeutic effect over time only with this formulation.

Given the claimed effect of Tz in triggering HER2 endocytosis, with subsequent downregulation of HER2 protein at the plasma membrane, we decided to specifically analyse the effect of Tz conjugated to NPs and free Tz on the expression level of the transmembrane pool of the receptor. We performed HER2 immunohistochemistry on tumour sections obtained from mice treated with the three different formulations. An immunoscore of HER2 expression from 0 to 3+ was assigned to each sample, as reported in Fig. 9b. Control tissue had 2+ score. We observed that at 5 h post injection, the HER2 immunoscore was comparable to that recorded in controls both in tumours treated with Tz and NP-Tz conjugates with 5NP-2Tz, while it decreased to 1 in case of treatment with NP-Tz conjugates with 5NP-1Tz. Over the following experimental times, no remarkable changes were observed in HER2 membrane expression in response to bifunctionalized NP-Tz conjugates. A decrease of the receptor immunoscore was recorded in Tz-treated tumours only between 24 and 48 h, while a further drop of HER2 expression up to 96 h occurred only in response to 5NP-1Tz. Selected immunohistochemical images of tumours isolated at 96 h post injection (Fig. 9c) clearly show that a sustained downregulation of HER2 expression in BC cell membrane is only evident in tumours treated with 5NP-1Tz.

The intracellular trafficking of HER2 on treatment with free or nanoconjugated Tz was also confirmed by immunofluorescence on tumour cryosections (Supplementary Fig. 18). It is intriguing to note that a significant trafficking of the receptor in BC cells occurred within the first 5 h post injection only in tumours treated with 5NP-1Tz, in line with the strong downregulation observed by immunohistochemistry at 5 h (Fig. 9b). All three Tz formulations altered HER2 membrane localization between 24 and 48 h post injection but, at 96 h, the HER2-associated fluorescence was restored on the plasma membrane of some Tz- and 5NP-2Tz-treated cells, according to the immumohistochemical images (Fig. 9c).

## Discussion

Generally speaking one might assume that receptor-mediated binding of ligand-modified NPs is improved by increasing the number of ligands per NP. However, there are certain restrictions to keep in mind. Concerning the NPs, in case of too high ligand density, accessibility of ligand to bind to receptors may be sterically hindered. In addition, higher ligand density may increase the size of the NP and thus NP diffusion to the receptor may be geometrically hindered in case of three-dimensional tissues. Concerning the cells, the receptor density of the plasma membrane is an important parameter. Only in case receptors can be close enough together to allow for multiple ligand binding from the ligands of one NP to several receptors, there may be an added benefit. Experimental quantification however is not trivial. This starts with the fact that determination of the number of ligands per NP is not straightforward, and that typically there is a large distribution of this number, that is, different NPs will have a different number of ligands bound per NP. Second, one has to distinguish between active and passive effects. Even without specific targeting, there is enrichment of NPs in tumours due to the EPR effect, which will overlay any active targeting effect.

In this work, we have developed a straightforward method for the synthesis of colloidal NPs functionalized with a discrete number of antibody molecules with a precise control on the ligand density on each NP. This was applied for antibodies specific to the target (Tz), and nonspecific antibodies (IgG) as control to probe for passive targeting. As expected, *in vitro* specific targeting was moderately increased for 5NP-2Tz versus 5NP-1Tz. Specificity could be demonstrated as NPs conjugated with nonspecific IgG antibodies showed negligible binding to cells. However, *in vivo* targeting was clearly improved for 5NP-1Tz versus 5NP-2Tz. Targeting was also less specific, as also NPs conjugated with nonspecific IgG antibodies were delivered to tumours, though at lower amounts. There is an ongoing discussion in literature about the possible contribution of active (that is, ligand–receptor-mediated) versus passive (that is, EPR effect-mediated) targeting of tumours with NPs. While in some studies clear contribution of active targeting is demonstrated, in other studies effects seem to be based predominantly on passive targeting^24^. Our results suggest that these discrepancies might be explained in terms of ligand density on the NP surface. In case of low ligand densities (in our study one specific antibody per each NP) contribution of active versus passive targeting seems to be higher than in case of higher ligand densities (in our study two specific antibodies per NP). We propose the hypothesis that a combination of the EPR effect and active targeting action is effective for 5NP-1Tz, in which the active targeting contribution becomes more important in proximity to the BC cells. In contrast, the EPR would be dominant for 5NP-2Tz. While there is indication for this hypothesis for our experimental conditions, the whole concept would have to be corroborated for different systems with controlled ligand density. Furthermore, our *in vivo* studies suggest that long-term intratumour retention of 5NP-1Tz contributes to a sustained therapeutic effect overtime as compared with 5NP-2Tz and with Tz in standard HER2-positive BC treatment. In this way, controlled conjugation of NPs with a defined ligand-NP stoichiometry, in particular monofunctionalized NPs, may lead to more efficient targeting strategies.

## Methods

### Reagents and instrumentation

All chemicals were purchased from Sigma-Aldrich (St Louis, MO) and used as received. Herceptin 150 mg was acquired from Roche. AFF660 dye was purchased from Life Technologies. Water was deionized and ultrafiltered by a Milli-Q apparatus from Millipore Corporation (Billerica, MA) before use. DLS measurements were performed with a Malvern Zetasizer. Viscosity and refractive index of pure water were used to characterize the solvent. NPs were dispersed in the solvent and sonicated in a S15H Elmasonic apparatus (Elma, Singen, Germany) before analysis. Final sample concentration used for measurements was typically of 0.2 μM.

### Synthesis of gold NPs

*Five-nanometre gold NPs*. Thiol-derivatized Au NPs were prepared by the Brust–Schiffrin method^16^ with some modifications, as described in detail in previous publications^18^. In brief, in a large separation funnel, a solution containing 2.17 g of tetraoctylammonium bromide in 80 ml of toluene was mixed with a solution containing 300 mg of tetrachloroauric acid in 25 ml of Milli-Q water. The organic phase was separated after several rounds of shaking and transferred in a large bowl. A solution containing 0.334 g of sodium borohydride in 25 ml Milli-Q water was added dropwise, resulting in a colour change to intense red, indicating formation of NPs, and left under vigorous stirring for 1 h. Afterwards, the resulting NP solution was washed with HCl and NaOH (25 ml, 10 mM) and Milli-Q water (25 ml, four times) while removing the aqueous phase after each step of washing. Finally, the resulting organic phase was left under stirring overnight. The day after, 10 ml of dodecanethiol was added and the solution heated at refluxed for 3 h at 65 °C. The solution was cooled down to room temperature, distributed in four separate vials, which were centrifuged at 550 *g* for 5 min. Non-aggregated NP solution was separated from the precipitated NPs and divided in six vials followed by dilution with methanol. Finally, the solutions were centrifuged for 5 min at 550 *g*, and the final NPs were redispersed in chloroform.

*Twelve-nanometre gold NPs*. Au NPs of ca. 12 nm core diameter were synthesized as reported by Schulz *et al*.^25^. A volume of 144 ml of Milli-Q water was added to 250 ml a round-bottomed flask and heated up until boiling with a heating plate. Then, sodium citrate (3.5 ml; 60 mM) and citric acid (1.5 ml; 60 mM) were added to the flask and kept under vigorous stirring for 30 min. Then 100 μl of ethylene diamine tetraacetic acid (EDTA, 30 mM) was added, followed by 1 ml of 25 mM hydrogen tetrachloroaurate (III) aqueous solution. After ca. 1 min the colour of the mixture became wine red. After the NP formation, the heating was stopped. When the temperature of the NP solution had lowered to 95 °C, the flask was immersed in ice to stop the reaction. To determine the concentration, the absorbance at 450 nm (extinction coefficient *ɛ*_450_=1.09 × 10^8^ M^–1^ cm^–1^) was used^26^.

*Twenty-nanometre gold NPs*. The 12 nm NPs were used as seed to NPs of 20 nm diameter. To do that the protocol described by Bastus *et al*.^27^ was followed. Seeds were heated up to 90 °C, and then 1 ml of HAuCl_4_ (25 mM) was injected. The solution was stirred for 30 min, and this process was repeated twice, waiting 30 min after each gold addition. After the NP growth the ultraviolet–visible spectrum was taken, and the concentration was determined using the absorbance at 450 nm (extinction coefficient *ɛ*_450_=5.41 × 10^8^ M^–1^ cm^–1^; extinction coefficient values extracted from Haiss *et al*.^26^.

### Phase transfer of gold NPs to organic solvent

Prior to the transference to organic solvent of the hydrophilic NPs, a ligand exchange with PEG is needed to provide them with enough stability. Thus, this process has two steps, which are explained below^28^.

*PEGylation*. The NP concentration was determined and the NPs were stabilized by mPEG−SH (CH_3_O-PEG-SH, *M*_w_=750 Da (Rapp Polymere)) dissolved in Milli-Q water. A volume of 10 μl of NaOH (1 M) per ml of GNPs was added to increase the pH to ca. 10, aiming to increase the reactivity of the thiol group. The stoichiometric ratio of PEG molecules to the NPs was 5 × 10^5^. The solution was mixed by stirring overnight, although for the size of these NPs the exchange is assumed to be complete within ca. 1 h (ref. 28).

*Phase transfer*. NPs were transferred to organic phase using a solution of dodecylamine in chloroform (0.4 M). Before this, PEGylated NPs were concentrated via centrifugation to a final volume of ca. 20 ml. Thus, 20 ml of dodecylamine solution were used for each. By using a vigorous stirring the NPs were transferred within 2 h. NPs were then cleaned via precipitation in, and washed twice with clean chloroform^28^.

### Phase transfer of gold NPs to aqueous solution

The NPs suspended in chloroform were transferred from organic to aqueous solution by wrapping an amphiphilic polymer around their surface, resulting in monodisperse and highly colloidally stable water-soluble NPs. An amphiphilic polymer, poly(isobutylene-*alt*-maleic anhydride)-graft-dodecyl (PMA) was synthesized by linking dodecylamine to 75% of the anhydride rings of polyisobutylene-*alt*-maleic anhydride (average *M*_w_ ∼6,000 g mol^–1^, Sigma, #531278)^18^,^29^,^30^. A fluorescent version of PMA was synthesized by linking FITC via its amine end to 1% of the anhydride rings of polyisobutylene-*alt*-maleic anhydride^18^. For the coating procedure an aliquot of *V*_p_=101 μl of *c*_p_=0.5 M PMA (concentration referring to the monomer units of the polymer, as dissolved in chloroform) was added to Au NPs in chloroform (*c*_NP_=2.27 μM, *V*_NP_=2 ml). The amounts of reagents were calculated based on the following equation: *V*_P_=(*π*·*c*_NP_·*V*_NP_·*d*_eff_^2^·*R*_p/area_)/*c*_P_. Hereby *c*_NP_ and *V*_NP_ are, respectively, the concentration and volume of the NP solution, *c*_P_ and *V*_P_ are, respectively, the concentration and volume of the polymer solution, *d*_eff_^2^ is the effective diameter of one NP and *R*_p/area_ is the number of polymer monomer units used per surface area. In this study *R*_p/area_=50 nm^–2^ was used (the *R*_p/area_ value for 12NP and 20NP was 3,000 nm^–2^). The mixture was homogenized and the solvent was then evaporated at reduced pressure. Sodium borate buffer (pH 12, 10 ml) was added obtaining a clean NP dispersion, which was concentrated in Amicon tubes (100 kDa filter cutoff) by centrifuging at 550*g*. Finally, the water-soluble polymer-coated NPs were washed twice diluting with H_2_O in the same way and concentrated to a final volume of 2 ml).

The core diameter^31^ of the Au NPs was determined with TEM and was found to be *d*_c_=4.8±1.3 nm (Supplementary Fig. 1). The core diameter for 12NP and 20NP was found to be *d*_c_=13.4±0.9 and 19.5±1.5 nm, respectively (Supplementary Fig. 11). We assumed in all the cases a thickness of the capping ligands of *l*_ligand_=1.8±0.2 nm. Thus, estimated an effective diameter of *d*_eff_=*d*_c_+2·*l*_ligand_. For 5NP *d*_eff_=8.4±1.7 nm (ref. 18).

The NP concentration was determined from the absorption at the maximum of the plasmon peak *A*=*l*·*c*_NP_·*ɛ* as measured in a cuvette with path length *l* and assuming a molecular extinction coefficient of the 5NPs of *ɛ*=8.63 × 10^6^ M^–1^ cm^–1^ (Supplementary Fig. 2). For 12NP and 20NP, the concentration was determined using the same method, but considering the absorbance at 450 nm and the following extinction coefficients: *ɛ*=1.09 × 10^8^ and 5.41 × 10^8^ M^–1^ cm^–1^, respectively (Supplementary Fig. 12)^26^. Note that the appropriate metrics for quantifying the amount of NPs are number concentrations (mol l^–1^] and not mass concentrations (g l^–1^). One NP with two antibodies attached has a much higher mass (considering the small diameter of the NPs) than one NP without any antibody attached. In case both samples would be added with the same mass concentration, there would be fewer NPs with two antibodies than NPs without antibody in solution, due to the higher molecular weight in the NPs with two antibodies attached per NP^27^. In the case of using solutions with the same number concentration, however, in both solutions the same number of NPs is present. For comparative studies to analyse the effect of NPs with different surface conjugation, therefore number concentrations (M) should be applied.

### Attachment of PEG to the surface of gold NPs

For the introduction of a discrete number of functional groups, standard bioconjugation chemistry using 1-ethyl-3-(3–dimethylaminopropyl)carbodiimide hydrochloride (EDC) was used exploiting the carboxylic groups on the surface of polymer-coated Au NPs. The 10 kDa diamine-PEG was selected as an optimal linker in-between the NPs and antibodies, because of its high molecular weight, which allowed us to clearly distinguish the distribution in discrete bands in the gel electrophoresis (Figs 1c and 2a). For all functionalization experiments, stock solutions of polymer-coated Au NPs with the concentration *c*_NP_=6 μM in 50 mM sodium borate buffer (pH 9.0) were prepared. The same buffer was used to dissolve the diamine–PEG (NH_2_–PEG–NH_2_) and EDC. For the coupling experiments, equal amounts of the NP solution (*c*_NP_=6 μM) and the PEG solution (*c*_PEG_=3 mM) were mixed and split into 20 μl samples. In all samples the ratio of PEG molecules to NPs was kept constant for all samples was *c*_PEG_/*c*_NP_=500. To these, 10 μl of an EDC solution of appropriate concentration was added to achieve ratios of EDC molecules to NPs of *c*_EDC_/*c*_NP_=32,000, 16,000, 8,000 and so on. With this series, conditions were established under which NPs with exactly two or with only one PEG molecule per NP could be best synthesized^21^. The large excess of PEG molecules (that is, NH_2_PEG–NH_2_) was chosen to prevent inter-particle crosslinking via the two amino groups of PEG. The samples were mixed with a pipette and allowed to react for at least 90 min. For analysis of the conjugation gel electrophoresis was applied^21^. Before running the gels, about 6 μl of gel-loading buffer containing bromophenol blue and 30% glycerol was added to each sample. Two per cent agarose gels were prepared with 0.5 × Tris/borate/EDTA buffer and run for 60–90 min at 10 V cm^–1^ (ref. 32). Analysis of these gels allowed for determining the EDC/NP ratio with the highest yield of Au NPs with one or two PEG molecules attached per NP (5NP-1P and 5NP-2P, respectively). For the preparation of a larger amount of 5NP-1P and 5NP-2P, a larger amount of *c*_NP_=6 μM polymer-coated Au NPs (*V*_NP_=450 μl) and consequently a large amounts of diamine–PEG (*c*_PEG_=3 mM, *V*_PEG_=50 μl) and an appropriate concentration of EDC were prepared in 50 mM sodium borate buffer (pH 9.0). After reaction the sample was loaded on a 2% agarose gel with a single big loading well for 90 min to separate NPs with a defined number of attached PEG molecules^10^. Because of the attachment of PEG, all reacted NPs showed a significant shift on the gel as compared with the bare NPs. In fact, the negatively charged Au NPs migrated towards the positive pole becoming more retarded the larger the number of PEG molecules attached, which permitted the isolation of 5NP-1P and 5NP-2P. The bands consisting of NPs with exactly one or two attached PEG molecules (and thus exactly one or two free amino groups at the PEG terminal pointing towards solution) were cut out and immersed separately in 0.5 × Tris/borate/EDTA buffer in a dialysis membrane (molecular weight cutoff (MWCO) 3,500 Da), and again an electric field was applied for 10 min, 10 V cm^–1^. The buffer containing the extracted NPs was collected and concentrated by centrifugation at 550*g* through 50 kDa Amicon centrifuge filter tubes.

### Conjugation of gold NPs with antibodies

The goal of this work was to set up a strategy to control the number and orientation of monoclonal antibodies covalently linked on the surface of individual NPs, giving rise to mono- and bi-Tz functionalized NP conjugates (5NP-1Tz and 5NP-2Tz, respectively). Our approach was characterized by the control of the number of functional binding sites (amino groups of attached PEG) on each NP to favour the introduction of one single active antibody (5NP-1Tz) or two antibodies (5NP-2Tz) covalently linked on the NP surface to compare their targeting efficiency. Most general conjugation strategies adopted for the immobilization of biological molecules on NPs are based on nonspecific approaches, in which biological molecules are passively adsorbed on the surface through electrostatic and hydrophobic interactions or covalently linked exploiting nondirectional coupling reactions^32^,^33^. In such cases, it is complicated to determine the number and orientation of targeting ligands on individual NPs. Therefore, virtually no structure–activity relationship data are available in the literature at present. In our approach, however, the attachment is directed and covalent.

Herceptin (15 mg ml^–1^) was purified from excipients by dialysis using Slide-A-lyzer cassette (MWCO 7,000 Da) in phosphate-buffered saline (PBS, EuroClone, pH 7.2) for 48 h at 4 °C. The yield of Tz was ∼30%. Purified Tz (1 mg) was labelled with Alexa Fluor 660 kit (Invitrogen). The labelled antibody solution was reconstituted with 1 ml of PBS (pH 7.2) at 4 °C. The carbohydrate unit of the vicinal rings in the Fc region of the Tz antibody were mildly oxidized with sodium periodate resulting in reactive aldehyde groups^4^. NaIO_4_ (0.1 mg; NaIO_4_/antibody in a 1:10 weight ratio) dissolved in 100 ml of PBS (pH 7.2) was added to the antibody solution and incubated for 30 min at 4 °C under shaking (protected from light). The antibody solution was transferred to an Amicon centrifuge filter tube (MWCO 10 kDa) and centrifuged for 10 min at 4 °C (3,000*g*). The concentrated antibody solution was diluted to 1 ml with PBS buffer (pH 7.2), loaded onto a PD-10 column (pre-equilibrated with PBS, pH=8) and centrifuged for 2 min at 1,000*g* (ref. 22).

The diluted antibody solution was added to each solution of 1 or 2 diamine–PEG functionalized NPs for 3 h at 4 °C under shaking. NaCNBH_3_ (0.01 ml) in PBS buffer (pH 8, 16 mM NaCNBH_3_) was added to each solution and incubated for 30 min at room temperature. The reaction of the NH_2_ terminal groups on 5NP-1P and 5NP-2P with the newly formed aldehyde functionalities in oxidized Tz resulted in an imine linkage, which was *in situ* reduced giving a stable secondary amine bridge^21^. To eliminate unreacted antibody, each functionalized NP solution was cleaned by dialysis for 1 h, 2 h and overnight in a Float-A-Lyzer Spectra/Por G2 (MWCO 300 kDa) under stirring, at 4 °C. As control, NP conjugates with generic rabbit IgG molecules instead of Tz antibody were synthesized in the same way, leading to 5NP-1IgG and 5NP-2IgG.

### Characterization of antibody conjugation

*Dynamic light scattering*. Each attached Tz or IgG molecule increases the overall size of a NP conjugate significantly, as, for example, human IgGs have intrinsic sizes of the same order of the NP (typically 9 × 14 nm). Hence, the occurred conjugation reaction could be monitored by DLS (Supplementary Fig. 3). DLS and laser Doppler anemometry measurements were carried out in water and hydrodynamic diameters *d*_h_, given as mean values from the number distribution, and *ζ*-potential values are summarized in Supplementary Table 1. The s.d. values were obtained from several independent measurements. Note that absolute size measurements with commercial DLS set-ups as used here always have to be interpreted carefully.

*Dot blot assay*. Dot blotting was performed by filtering proteins and/or NPs onto polyvinylidene fluoride membranes, utilizing a Manifold I dot blot apparatus (GE Healthcare). Then each polyvinylidene fluoride membrane was incubated in blocking solution (5% skim milk in PBS, Tween 0.05%) for 1 h at room temperature and probed for 1 h at room temperature in blocking solution using goat anti-human-horseradish peroxidase antibody (Tebu-bio) at a 1:18,000 dilution. Membranes were rinsed three times in 0.05% Tween in PBS for 10 min. Immunoreactive spots were revealed using ECL western blotting reagent (GE Healthcare) and acquired with Odyssey Fc reader (LI-COR Biosciences). Dot blot analysis confirmed the occurrence of Tz binding to the PEG chains and the maintenance of a proper protein folding and activity following conjugation (Supplementary Fig. 4).

*Fluorescence assay*. Fluorescence analyses were performed to check the presence of antibodies on the NP surface and afterwards to investigate the maximum number of antibodies on each NP. In particular, we explored also the possibility to isolate NP conjugates bearing three or more antibodies with the aim to determine the maximal number of accessible targeting ligands on each individual NP. Therefore, a batch of NPs containing three PEG ligands (5NP-3P) was prepared and isolated according to the same procedure as used for 5NP-1P and 5NP-2P by increasing the EDC/NP ratio in the reaction mixture. Fluorescence spectra were recorded using a Fluoromax-4P spectrofluorimeter from Horiba Scientific (NJ, USA). Samples were excited at a fixed wavelength (*λ*_ex_=663 nm) and spectra were recorded in a wavelength range between 673 and 800 nm. The fluorescence emission of fluorescently labelled Tz (Tz-Alexa_660_) was detected at 690 nm. The slit widths (for controlling magnitude and resolution of transmitted light) were standardized at 5 and 5 nm for excitation and emission wavelength, respectively. The data are shown in Fig. 2b. Fluorescence rises on attaching two instead of one Tz per NP, thus demonstrating the different amount of Tz for the 5NP-1Tz and 5NP-2Tz conjugates. However, the conjugation with three molecules of Tz was not possible as resulted from the comparison of the fluorescent intensity of 5NP-1Tz, 5NP-2Tz and 5NP-3Tz, measured after reacting 5NP-1P, 5NP-2P and 5NP-3P, respectively, with dye-labelled Tz (Fig. 2b). Indeed, the FI of 5NP-3Tz was essentially the same as that of 5NP-2Tz, suggesting a saturation of the NP surface area right after the introduction of two antibodies.

*NP incubation with biological fluid*. A unit of 4.6 pmol of each functionalized NP solution was incubated in 1 ml of a 0.4 mg ml^–1^ fetal bovine serum (FBS) solution for 5 and 48 h at 4 °C under shaking. After incubation time both solutions were centrifuged at 5,500*g* for 15 min at 4 °C to precipitate the NP-hard corona complex^34^, and supernatants were removed using a syringe. Precipitates were resuspended in PBS buffer (pH 7.2).

*Identification of the protein corona by SDS–PAGE*. Functionalized NP-hard corona complexes obtained as explained above were added to loading buffer (62.5 mM Tris-HCl (pH 6.8), 2% (w/v) SDS, 10% glycerol, 0.04 M dithiothreitol and 0.01% (w/v) bromophenol blue) in PBS and heated at 100 °C for 10 min. Samples were loaded on SDS–PAGE^35^ carried out in a Mighty Small apparatus (Hoefer Scientific Instruments, San Francisco, CA) with an 8% acrylamide running gel and a 4% stacking gel, 90 min at 25 mA. Proteins were revealed by Imperial Protein Stain (Thermo Scientific). Data are shown in Supplementary Fig. 5.

*BSA labelling using FITC*. To further evaluate the effect of the immobilization of different number of ligands on the NP surface, the amount of protein corona after incubation of 5NP-1Tz and 5NP-2Tz with BSA was examined. For this purpose, first BSA had to be labelled with FITC. A volume of 0.2 ml of 25 mM FITC was added to 0.2 nmol of BSA (Sigma-Aldrich) dissolved in 0.8 ml of 0.1 M NaHCO_3_, pH 8.35. The reaction solution was vigorously stirred at room temperature for 2 h. To eliminate FITC excess, the solution was loaded onto a PD10 column. Afterwards different samples including different concentrations of BSA-FITC were prepared to obtain a calibration curve. Fluorescence spectra were recorded using a Fluoromax-4P spectrofluorometer. Samples were excited at a fixed wavelength (*λ*_ex_=488 nm) and spectra were recorded in a wavelength window between 498 and 700 nm. The fluorescence emission of BSA-FITC was detected at 518 nm. The slit widths (for controlling magnitude and resolution of transmitted light) were standardized at 5 nm for both excitation and emission wavelength.

*Incubation of functionalized NPs with BSA-FITC and quantification of BSA-FITC corona by fluorescence assay*. A unit of 4.6 pmol of each functionalized NP (5NP-1Tz and 5NP-2Tz) were incubated in 1 ml of a 0.4 mg ml^–1^ BSA-FITC solution for 5 and 48 h at 4 °C under shaking. After incubation time, both solutions were centrifuged at 5,500*g* for 15 min at 4 °C to precipitate NP-BSA-FITC corona complex, and supernatants were removed using a syringe. Precipitates were resuspended in PBS buffer (pH 7.2) and analysed with a spectrofluorometer. As expected, the surface functionalization decreased the extent of protein adsorption as it results from the measurements of FI, which, in the case of 5NP-1Tz, was twofold higher than 5NP-2Tz (Supplementary Fig. 6). In this way, the results confirm a double-accessible surface area in 5NP-1Tz compared with 5NP-2Tz due to the different extent of functionalization.

*NP incubation with biological fluid and DLS analysis of stability*. A unit of 4.6 pmol of one or two Ab-functionalized NPs solution was incubated in 1 ml of a 0.4 mg ml^–1^ FBS solution up to 96 h at 4 °C under shaking. At 5, 24, 48, 96 h of incubation both solutions were analysed by DLS.

*Fluorescence quenching experiments*. To determine if the fluorescence of the NPs can be altered *in vivo*, 5NP-1P, 5NP-2P, 5NP-1Tz and 5NP-2Tz were incubated with human plasma at the same concentration. A volume of 50 μl of 0.1 mg ml^−1^ of all the samples were diluted in 200 μl of human plasma (obtained with consensus from 35 old male). Aliquots of 50 μl were taken at times 1, 2, 8, 24 and 48 h. The fluorescence was recorded at different time points exciting at 490 nm. The emission at 515 nm was represented for all the samples and time points (Supplementary Fig. 19). The emission of 5NP-1P and 5NP-2P can be neglected, as it is comparable to the emission collected just using the plasma as sample (Supplementary Table 3). Fluorescence of 5NP-1Tz and 5NP-2Tz remains constant along time with variations of a 10% as maximum, as it is shown in the normalized data (Supplementary Table 4). Remarkably, the presence of one or two antibodies can be confirmed in all the cases based on the fluorescence (Supplementary Table 3), that is, 5NP-2Tz has the double fluorescence than 5NP-1Tz.

### Cell cultures

JMT-1 was purchased by AddexBio (San Diego, CA), while the other cell lines were purchased by American Type Culture Collection. MCF-7, MDA-MB 453 and MDA-MB 468 cells were cultured in 50% Dulbecco's modified Eagle's medium (DMEM) high glucose and 50% Ham's F-12 nutrient mixture, supplemented with 10% FBS, L-glutamine (2 mM), penicillin (50 UI ml^–1^) and streptomycin (50 mg ml^–1^), while BT474 and SKBR3 cells were cultured in RPMI 1640 medium, supplemented with 10% FBS, L-glutamine (2 mM), penicillin (50 UI ml^–1^) and streptomycin (50 mg ml^–1^). MDA-MB 231 cells were cultured in MEM Medium, supplemented with 10% FBS, L-glutamine (2 mM), penicillin (50 UI ml^–1^) and streptomycin (50 mg ml^–1^), while JIMT-1 were cultured in DMEM medium, supplemented with 10% FBS, L-glutamine (2 mM), penicillin (50 UI ml^–1^) and streptomycin (50 mg ml^–1^). All cell lines were tested grow at 37 °C and 5% CO_2_ in a humidified atmosphere and were subcultured before confluence using trypsin/EDTA. Cell culture medium and chemicals were purchased from EuroClone (Italy).

### HER2 expression

MDA-MB 231, JIMT-1, MCF-7, MDA-MB 453, SKBR3, BT474 and MDA-MB 468 cells (5 × 10^5^) were immunodecorated in fluorescence-activated cell sorting tubes with Tz (Herceptin, Roche, 1 μg per tube in PBS, 2% BSA and 2% goat serum) for 30 min at room temperature. Then, cells were washed thrice with PBS and immunodecorated with Alexa Fluor 488 goat anti-human secondary antibody (1 μl per tube in PBS, 2% BSA and 2% goat serum) for 30 min at room temperature. After three washes with PBS cells were analysed by Cytoflex flow cytometer (Beckman Coulter). In all, 10,000 events were acquired for each analysis, after gating on viable cells. A sample of cells immunodecorated only with secondary antibody was used to set the region of positivity.

### Cell-binding assay

MCF-7, BT474 and MDA-MB 468 cells were seeded day before at a concentration of 3 × 10^5^ cells per well. Then, cells were incubated 1 h at 37 °C in culture medium supplemented with 13 and 65 nM FITC-labelled 5NP-1Tz and 5NP-2Tz. FITC-labelled 5NP-1IgG and 5NP-2IgG or 5NP-1 and 5NP-2 were used as specificity controls. After incubation, cells were washed three times with PBS. Labelled cells were resuspended in 0.5 ml of PBS and analysed by Cytoflex flow cytometer (Beckman Coulter). A total of 10,000 events were acquired for each analysis, after gating on viable cells and on singlets. A sample of untreated cells was used to set the appropriate gates.

### Confocal laser scanning microscopy and immunofluorescence

To assess co-localization with different intracellular markers, MCF-7 cells were cultured on collagen pre-coated coverglass slides, until sub-confluence and were incubated 1 and 4 h at 37 °C with 65 nM of NP-1Tz and NP-2Tz. Then, cells were washed twice with PBS, fixed for 10 min with 4% paraformaldehyde and treated for immunofluorescence. After fixation with 4% paraformaldehyde, cells were washed with PBS and then treated for 5 min with 0.1% Triton X-100. A blocking step was performed for 1 h at room temperature with a solution containing 2% BSA, 2% goat serum and 0.2 μg ml^−1^ DAPI (4′,6-diamino-2-phenylindole) in PBS. Golgi apparatus, lysosomes, early endosomes and recycling endosomes were stained, respectively, with Golgi Marker-130 (GM-130; cod. 610823, 1:100 dilution; clone 35; BD Biosciences), antibodies against cathepsin D (CatD; cod. IM03-100 μg 1:50; clone BC011; Millipore), early endosome antigen-1 (EEA1; cod. 610457; 1:1,000; clone 14; BD Biosciences) and transferrin (Tf; ab114008; 1:100; clone 5G2; Abcam) by incubating 2 h at room temperature and revealed by a Alexa Fluor 546-conjugated antibody against murine IgGs (A-11030; Invitrogen) at a 1:300 dilution by incubating for 2 h at room temperature in PBS, 2% BSA and 2% goat serum. Cells were mounted in Prolong Gold antifade reagent (Molecular Probes). All chemicals used in the preparation of samples for confocal microscopy were purchased from Sigma-Aldrich (Milano, Italy), except for DAPI, and the primary and secondary antibodies purchased from Life Technologies Italia (Monza, Italy). Microscopy analyses of stained cells were then performed with a Leica TCS SPE confocal microscope (Leica Microsystems, Wetzlar, Germany) at Fondazione Filarete, Milano, Italy. Images were acquired at 1,024 × 1,024 pixel resolution and with a × 63 or a × 40 magnification oil-immersion lens for cells or sections, respectively. Confocal microscopy images were analysed with Image J to calculate Pearson coefficient values with the JaCoP plugin.

### Cell viability assay

MCF-7 cells were cultured on a 96-multi-well dish at the density of 5,000 cells per square cm. Then, cells were incubated with different amounts of 5NP-1Tz and 5NP-2Tz (13 and 65 nM). Untreated cells and Tz-treated cells (13 and 65 nM) were used as controls. At the indicated time points (24, 48 and 72 h), cells were washed with PBS and then incubated for 3 h at 37 °C with 0.1 ml of 3-(4,5-dimethyl-2-thiazolyl)- 2,5-diphenyl-2H-tetrazolium bromide (MTT) stock solution previously diluted 1:10 in DMEM medium without phenol red. After incubation, MTT solubilizing solution (0.1 ml) was added to each well to solubilize the MTT formazan crystals (Promega). Absorbance was read immediately in a microplate reader (BioTek) using a testing wavelength of 570 nm and a reference wavelength of 620 nm. The results are normalized on viability of untreated samples and expressed as means±s.e. of six individual experiments.

### Cell death assay

MCF-7 cells were cultured on a 12-multi-well dish until sub-confluence. Then, cells were incubated 3 and 24 h at 37 °C in the presence of different amounts of 5NP-1Tz and 5NP-2Tz (13 and 65 nM). Untreated and Tz-treated cells (13 and 65 nM) were used as controls. After incubation, cells were washed twice with PBS and treated for fluorescence-activated cell sorting analysis according to the Annexin V-PE-Cy5 Apoptosis Detection Kit manufacturer's protocol (BioVision). Briefly, cells were resuspended in Binding Buffer and incubated for 5 min in presence of 5 μl of Annexin V-PE-Cy5. Cells were analysed within 1 h on a Cytoflex flow cytometer (Beckman Coulter). In all, 20,000 events were acquired for each analysis, after gating on viable cells. For evaluation of late apoptosis, the same protocol was used but incubation with 7- aminoactinomycin D (BD Biosciences; 51-68981E; 5 μl per sample) was accomplished.

### Production of xenograft tumour models

All animal experiments were conducted under an approved protocol of the Italian Ministry of Health. Animals were cared according to the guidelines of the Italian Ministry of Health and in the total respect of EU guidelines for the animal welfare. MCF-7 cells, grown as described above, were injected in 8-week-old female Balb/c nude mice after insertion on the neck of oestrogen pellets (Innovative Research of America, USA) by using a trocar. All tumour injections were done 2 days after pellet placing. MCF-7 (10^7^ cells for each animal) were suspended in growth medium and mixed with Matrigel high factor (Sacco Srl, Italy) in 3:1 ratio and injected into mammary fat pad of mice. Animals were observed and tumour formation was recorded at least three times per week. Tumours were allowed to grow up to 8 mm in diameter before mice treatment.

### Measurement of NP gold in mice plasma

5NP-1Tz or 5NP-2Tz (18 pmol per g body weight), or 12NP or 20NP (3 μg g^−1^) were injected in the tail vein of mice, and retro-orbital blood collection was performed at 5, 24, 48 and 96 h post injection. Collected blood was immediately added to heparin (Enoxaparin sodium 8000 IU anti-Xa activity) in a ratio 4:1 and then centrifuged (10 min at 3,000*g*). Plasma was then recovered and stored at –20 °C.

### Tumour targeting

AF660-labelled NP-Tz conjugates (18 pmol per g body weight) or Tz (18 pmol per g body weight), or 12NP or 20NP (3 μg g^−1^) were injected in the tail vein of mice and Epf imaging was performed at 5, 24, 48, and 96 h post injection by placing the animals, anaesthetized by intraperitoneal injection of 20 mg ml^–1^ of Avertin, in an IVIS Lumina II fluorescence imaging system (Calipers Life Sciences, UK) at 37 °C. Images were acquired with a Cy5 emission filter while excitation was scanned from 570 to 640 nm, and mice autofluorescence was removed by spectral unmixing. After *in vivo* acquisitions, mice were killed, and dissected tumours were analysed in the IVIS system as described above for the whole animals. All Epf values of tumour ROI and of the isolated tissues were normalized to the Epf obtained by IVIS acquisition of the injected solutions in a 96-well plate ( × 10^4^), using the same acquisition parameters. The FI of tumour homogenates was also measured. According to the procedure, the isolated tissues were weighted and homogenized with ultraturrax in a homogenization buffer (0.32 M sucrose, 100 mM HEPES, pH 7.4). Protein concentration of the samples was measured using the bicinchonic acid (Pierce, CA) protein assay, while fluorescence was analysed in a GloMax Multi Detection System (Promega). The fluorescence values of all homogenates were normalized on the FI of the injected solutions ( × 10^5^) as measured using the same spectrofluorimetric parameters.

### Immunohistochemistry of *ex vivo* specimens

The 3 μm sections from formalin-fixed and paraffin-embedded tissues were cut, deparaffinized in xylene and rehydrated in ethanol. Immunohistochemistry was performed, after microwave oven pretreatment (pH 8.0, EDTA buffer, 2 × 50 min), by using a polyclonal antibody rabbit anti-653 human c-erb-2 oncoprotein (1:1,000 dilution, DakoCytomation, 2 h incubation). The reaction was revealed by means of supersensitive non-biotin detection system (BioGenex) and diaminobenzidine as chromogen. To quantify c-erb-2 expression, a semiquantitative score based on the extension and intensity of immunohistochemical staining was applied (score from 0 to 3+). Data were analysed by GraphPad Prism software.

### Confocal laser scanning microscopy and immunofluorescence

To assess the co-localization of nanoconjugates with different intracellular markers, MCF-7 cells were cultured on collagen pre-coated coverglass slides, until sub-confluence and were incubated 1 and 4 h at 37 °C with 65 nM of NP-1Tz and NP-2Tz. Then, cells were washed twice with PBS, fixed for 10 min with 4% paraformaldehyde and treated for immunofluorescence. After fixation with 4% paraformaldehyde, cells were washed with PBS and then treated for 5 min with 0.1% Triton X-100. A blocking step was performed for 1 h at room temperature with a solution containing 2% BSA, 2% goat serum and 0.2 μg ml^−1^ DAPI in PBS. Golgi apparatus, lysosomes, early endosomes and recycling endosomes were stained, respectively, with Golgi Marker-130 (GM-130; cod. 610823, 1:100 dilution; clone 35; BD Biosciences), antibodies against cathepsin D (CatD; cod. IM03-100 μg 1:50; clone BC011; Millipore), early endosome antigen-1 (EEA1; cod. 610457; 1:1,000; clone 14; BD Biosciences), Tf (ab114008; 1:100; clone 5G2; Abcam) by incubating 2 h at room temperature and revealed by a Alexa Fluor 546-conjugated antibody against murine IgGs (A-11030; Invitrogen) at a 1:300 dilution by incubating for 2 h at room temperature in PBS, 2% BSA and 2% goat serum. Cells were mounted in Prolong Gold antifade reagent (Molecular Probes). All chemicals used in the preparation of samples for confocal microscopy were purchased from Sigma-Aldrich (Milano, Italy), except for DAPI, and the primary and secondary antibodies purchased from Life Technologies Italia (Monza, Italy).

For the confocal microscopy of cryosections, MCF-7 tumours were isolated and fixed in 4% paraformaldehyde solution for 3 h, washed in PBS and embedded in frozen tissue matrix (OCT) for freezing in liquid nitrogen. The 10 μm-thick tumour cryosections were air-dried at room temperature for 1 h, rinsed with PBS and treated for immunofluorescence. For the immunodecoration of HER2 and cytokeratin19, tumour cryosections were permeabilized with 0.1% Triton X-100 in PBS for 5 min. A blocking step was then performed for 1 h at room temperature with a solution containing 2% BSA and 2% goat serum in PBS. Then, we performed a 2 h incubation with the same rabbit anti-human c-erb-2 used for the immunohistochemistry (dilution 1:1,000) or with a polyclonal antibody rabbit anti- cytokeratin19 (Abcam; dilution to 5 μ ml^−1^). After three times washing with PBS, the primary antibody was revealed by an Alexa Fluor 488-conjugated antibody against rabbit IgGs at a 1:300 dilution, in a 2% BSA, 2% goat serum solution and DAPI (1:1500) for 2 h at room temperature.

Microscopy analyses of stained cells and cryosections were then performed with a Leica TCS SPE confocal microscope (Leica Microsystems, Wetzlar, Germany) at Fondazione Filarete, Milano, Italy. Images were acquired at 1,024 × 1,024 pixel resolution and with a × 63 or a × 40 magnification oil-immersion lens for cells or sections, respectively. Confocal microscopy images of cultured cells were analysed with Image-J to calculate Pearson coefficient values with the JaCoP plugin.

### TEM of *ex vivo* specimens

TEM analyses of NPs were performed by means of a transmission electron microscope (Zeiss EM109), prepared by slow evaporation of one drop of aqueous solution of the NPs placed on a formvar/carbon-coated copper mesh grid and air-dried. For *ex vivo* analyses, small portions of MCF-7 tumour samples were fixed in 2.5% glutaraldehyde in 0.1 M phosphate buffer, pH 7.2, for 2 h. After one rinsing with phosphate buffer, specimens were post-fixed in 1.5% osmium tetroxide for 2 h, dehydrated by 70, 90 and 100% ethanol, and embedded in epoxy resin (PolyBed 812 Polysciences Inc., USA). Ultrathin sections were examined by the Zeiss EM109 microscope.

### Quantification of gold in plasma by ICP-MS

ICP-MS analysis to determine the amount of Au found in the different organs of mice after injection of Au NPs was carried out similar to previous reports^20^. Before the measurement took place, the ICP-MS set-up was calibrated with a freshly prepared serial dilution of gold (Roth, Au-Standard (1,000 mg ml^−1^). The used calibration curve was constructed using gold concentrations from 10 p.p.b. (parts per billion) to 2,500 p.p.b. In addition, the auto-tuning solution from Agilent for ICP-MS 7500cs with a standard concentration of 1 μg l^−1^ of Ce, Co, Li, Mg, Tl and Y was used to set the general background, as well as to calibrate the electrical field of the lenses and the quadrupole field in strength and frequency. Also accounted for were oxidation of the ionized species during the tuning as well as double charge occurrences. In the so calibrated set-up, the oxidation species rate was lower than 1.0% and double charge rate was below 2%. All vials and working materials were either cleaned using freshly prepared aqua regia for 2 h followed by boiling in Milli-Q water, or were sterile and clean non-reusable consumables. The samples were introduced into the ICP-MS set-up through a perfluoroalkoxy-alkane-based microflow spray chamber, where the aqueous sample was nebulized, introduced into the argon gas flow and transported to the torch, where it was ionized in an argon plasma of around 6,000 °C. After ionization, the sample was presorted using an omega lens, element-wise separated in a quadrupole field through the mass to charge rate, again sorted using kinetic barriers and a charged lens system, and finally detected with either an analogue or a digital detector depending on the count rate.

The extracted mouse organs with a weighted mass of *m*_Organ_ were dissolved before measurement using 67 wt% HNO_3_ with a volume *V*_HNO_3__=2 ml (Fisher Chemical, #7697-37-2) per organ for 48 h under constant agitation at room temperature. From the solution containing the digested organ in HNO_3_, 200 μl were taken for analysis, mixed with 400 μl 37 wt% HCl (Fisher Chemical, #7647-01-0), that is, diluted by a factor 3, to enhance the digestion of the incorporated Au NPs and prepare for the ionization process during the plasma interaction. This solution was again diluted by a factor of 10 with 2 wt% HCl after 2 h of digestion to protect the ICP-MS set-up from the aqua regia, resulting in an overall dilution factor *α*_dil_=30. This diluted solution was then measured three consecutive times to determine the mass concentration *C*′_Au_ of elemental gold in solution. *C*′_Au_ (μg l^−1^) describes the solutions concentration. μg/L is also referred to as p.p.b. (or 1 μg kg^−1^ (solution)=10^−9^ g g^−1^ (solution)), as the density of all used solutions is equal to the one of water=1 kg l^−1^. Δ*C*′_Au_ (%) describes the s.d. between the three measurements. Thus, the concentration in the original solution with the digested organ was calculated to *C*_Au_ (p.p.b.)=*C*′_Au_·*α*_dil_ (in a previous work^20^ the results were presented as *C*_Au_/*m*_Organ_ (p.p.b. g^−1^), whereby the scaling factor to *m*_Au_/*m*_Organ_ was *V*_HNO3_). The total mass of gold in each organ was calculated as *m*_Au_ (g)=*C*_Au_·*V*_HNO3_. Thus, the mass of gold per mass of organ is *m*_Au_/*m*_Organ_. Data are provided in Supplementary Table 6 and Supplementary Fig. 9.

### HER2 expression in *ex vivo* specimens

HER2 expression in MCF-7 tumour lysates was assessed after isolation at 5, 24, 48 or 96 h of exposure to 5NP-Tz, 5NP-2Tz or free Tz (Supplementary Fig. 16). Tumours were lysed in SDS–PAGE application buffer, electrophoresed and immunoblotted using anti-HER-2 antibody. Calnexin expression was assessed as control. Uncropped scan of the original immunoblot is reported in Supplementary Fig. 17.

### Data availability

The authors declare that the data supporting the findings of this study are available within the paper and its Supplementary Information files.

## Additional information

**How to cite this article:** Colombo, M. *et al*. Tumour homing and therapeutic effect of colloidal nanoparticles depend on the number of attached antibodies. *Nat. Commun.* **7,** 13818 doi: 10.1038/ncomms13818 (2016).

**Publisher's note:** Springer Nature remains neutral with regard to jurisdictional claims in published maps and institutional affiliations.

## Supplementary Material

Supplementary Information Supplementary Figures, Supplementary Tables.
