# Supplementary material for: Tumour homing and therapeutic effect of colloidal nanoparticles depend on the number of attached antibodies
Source: Nat Commun. 2016 Dec 19;7:13818. doi: 10.1038/ncomms13818 (PMC5187442; doi:10.1038/ncomms13818)
Supplement: Supplementary Information — Supplementary Figures, Supplementary Tables. [file ncomms13818-s1.pdf]

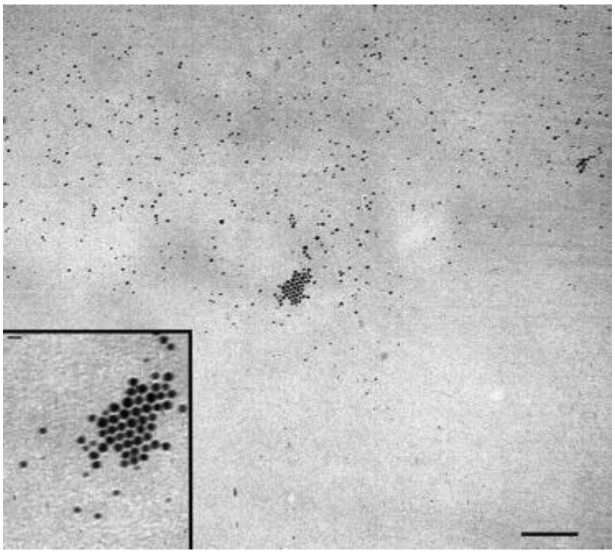

Supplementary Figure 1. TEM image of 5NP (the scale bar corresponds to 50 nm and 5 nm in the magnification).

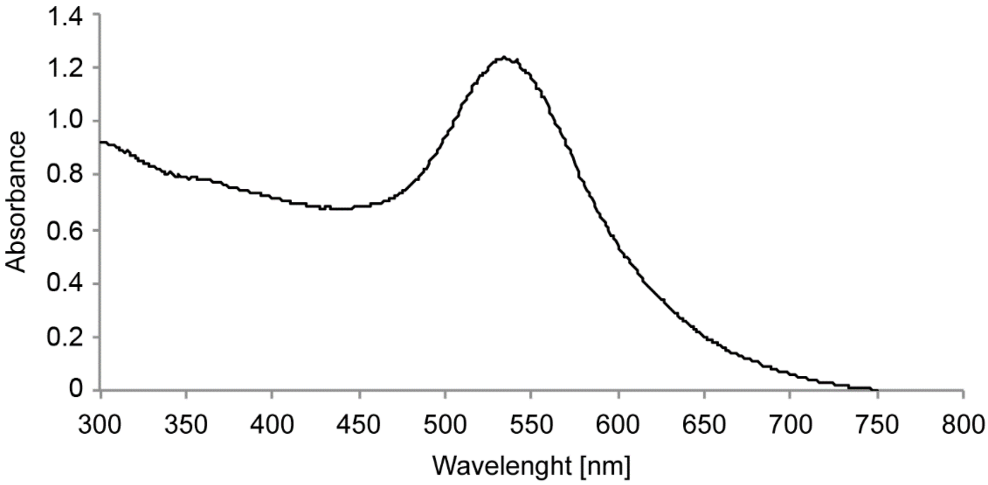

Supplementary Figure 2. Absorption spectrum A(λ) of polymer-coated 5NPs as dissolved in water.

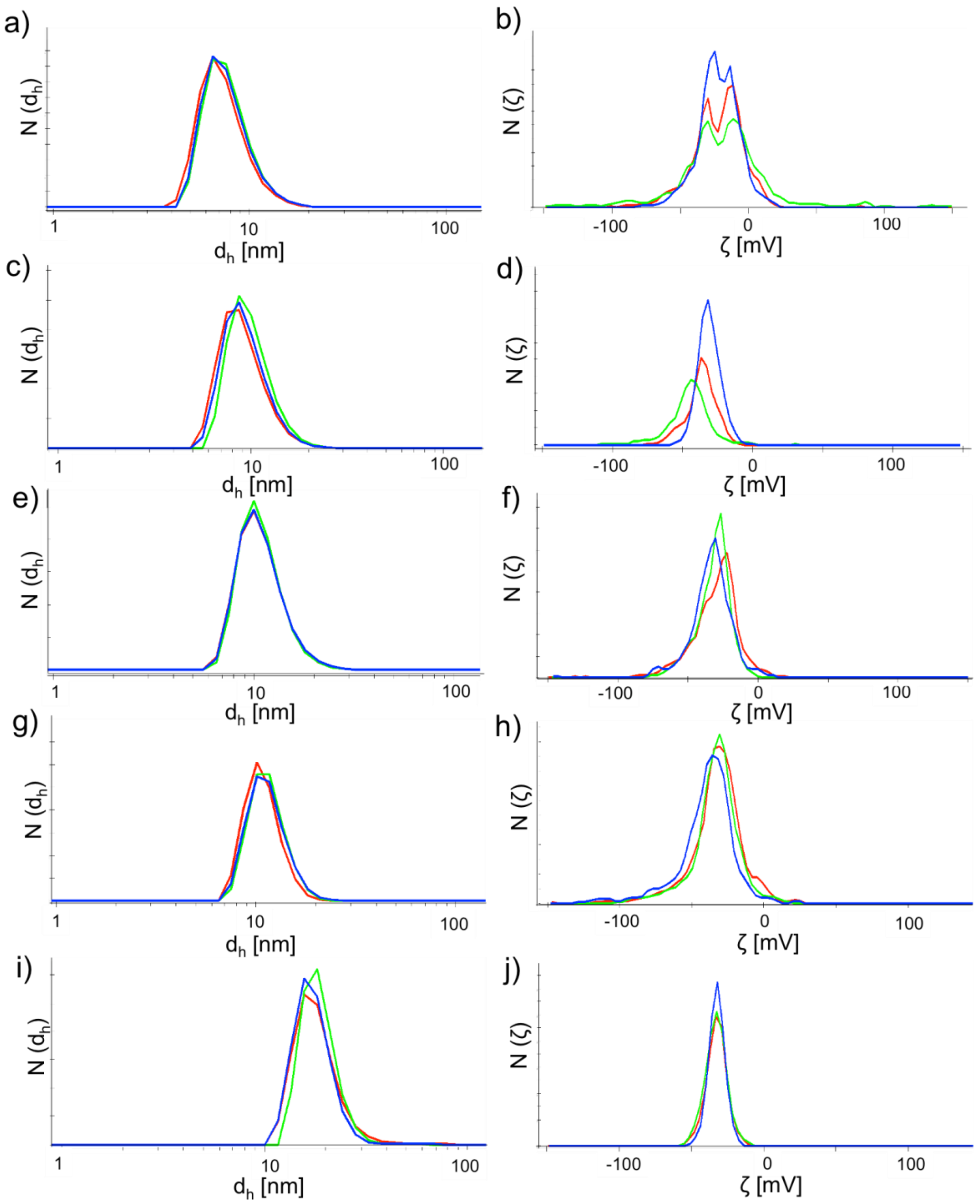

Supplementary Figure 3. Distribution of hydrodynamic diameters N(d_h) of NPs, as determined from the number distribution in water of a) 5NPs, c) 5NP-1P; d) 5NP-2P; g) 5NP-1Tz; i) 5NP-2 Tz; and distribution of ζ-potential N(ζ) of NPs, as determined of the zeta-potential (ζ) in water of b) NPs, d) 5NP-1P; f) 5NP-2P; h) 5NP-1Tz; j) 5NP-2Tz.

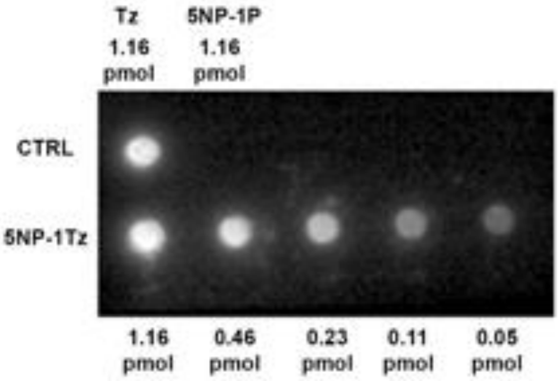

Supplementary Figure 4. Assessment of Tz conjugation on 5NP-1P. Decreasing amounts of monofunctionalized Au NPs were loaded on a PVDF membrane. Trastuzumab (Tz) and 5NP-1P were used as positive and negative controls (CTRL), respectively. The presence of Tz was detected by anti-human Horse Radish Peroxidase-antibody only in 5NP-1P spots and in positive control (Tz), confirming that Tz was linked to 5NPs.

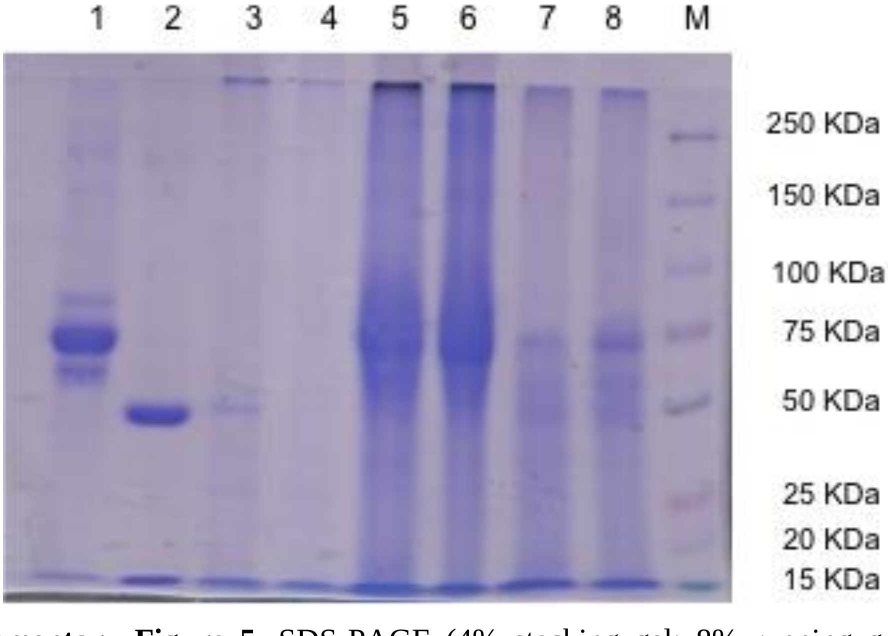

Supplementary Figure 5. SDS-PAGE (4% stacking gel; 8% running gel). 1) Fetal Bovine Serum (FBS); 2) Tz; 3) 5NP-2Tz; 4) 5NP-1Tz; 5) 5NP-2Tz after 48 h incubation with FBS; 6) 5NPs-1Tz after 48 h incubation with FBS; 7) 5NPs-2Tz after 5 h incubation with FBS; 8) 5NPs-1Tz after 5 h incubation with FBS. M) Marker; lanes 1-5-6-7-8 show that major representative protein is bovine serum albumin (BSA, 66.5 kDa).

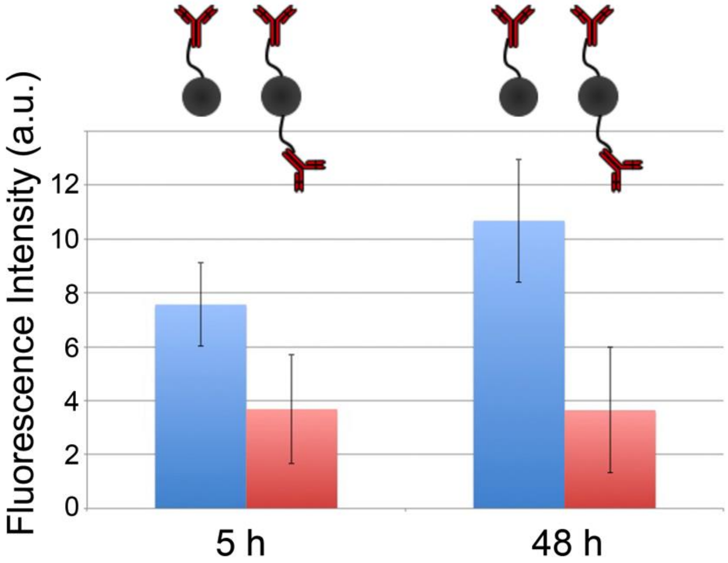

Supplementary Figure 6. Fluorescence intensity of NP conjugates (5NP-1Tz and 5NP-2Tz), which have been incubated with FITC-labeled BSA.

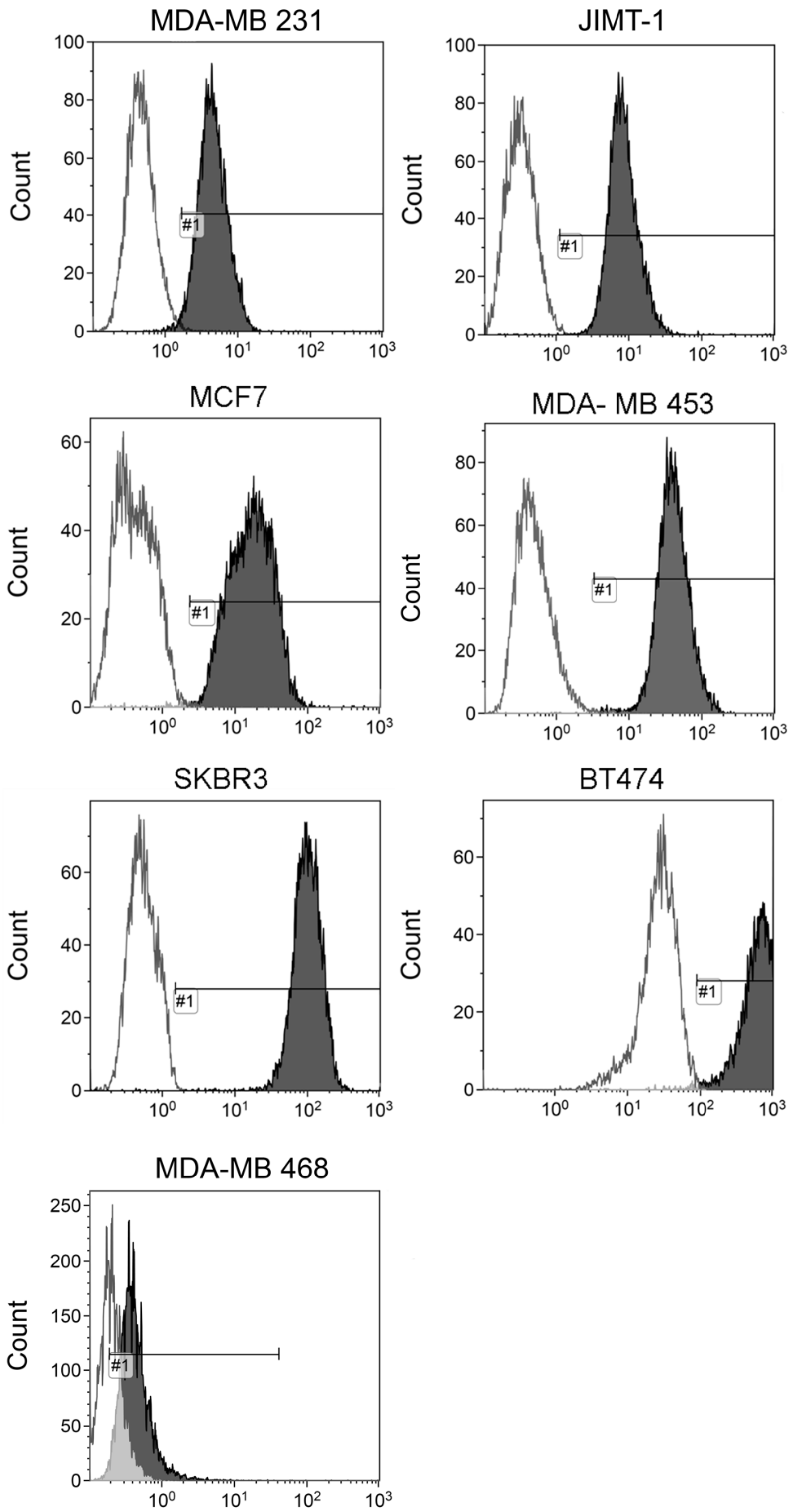

Supplementary Figure 7. Flow cytometry analysis of HER2 expression in different human breast cancer cells. Seven human breast cancer cell lines with HER2 expression basal (MDA-MB 231 and MDA-MB 468), moderate (MCF7, MDA-MB 453 and JIMT-1) or high (SKBR3 and BT474) have been examined. Cells immunodecorated with the antihuman secondary antibody conjugated with AlexaFluor 488 were used to set the gate on viable cells and the region of positivity.

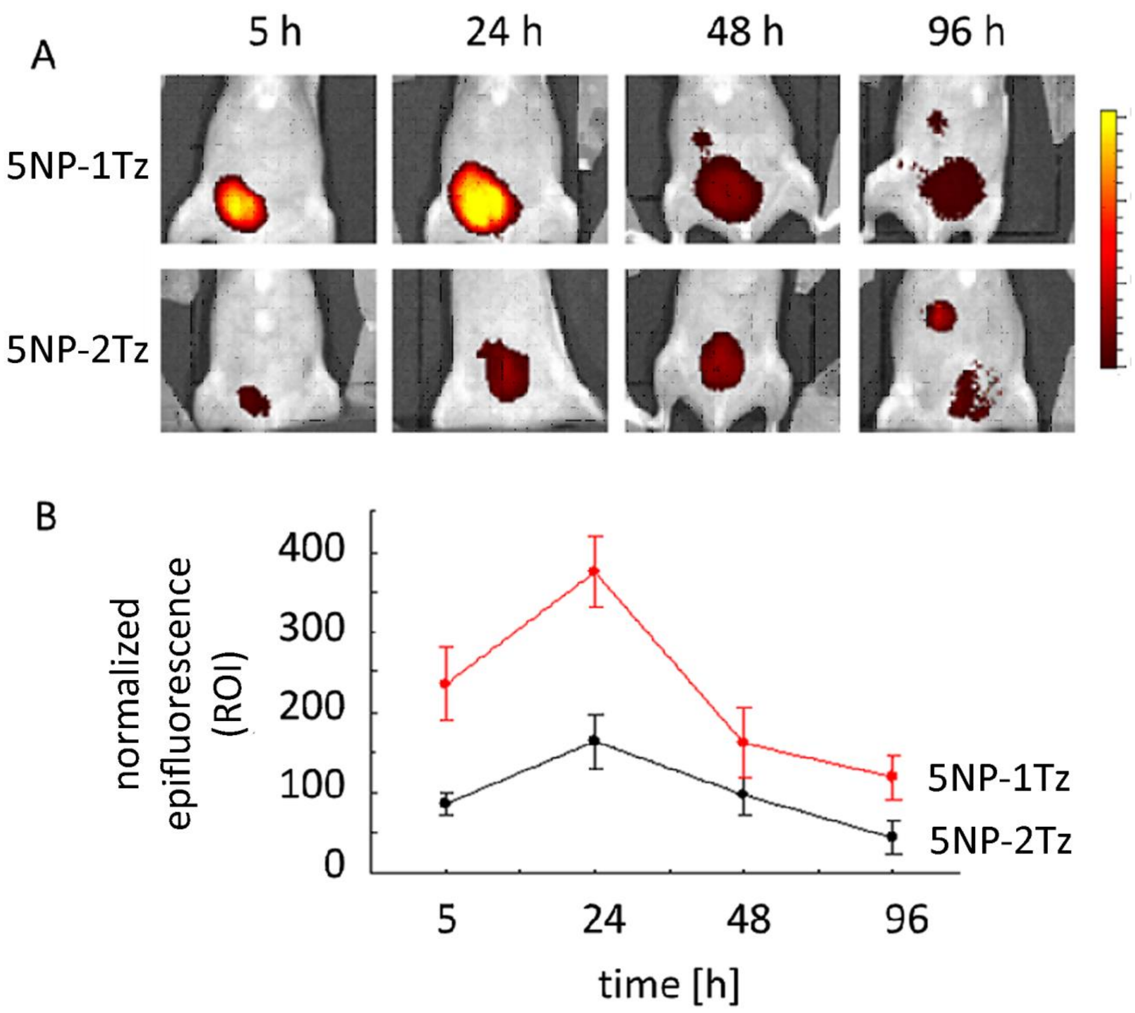

Supplementary Figure 8. (A) Epifluorescence (Epf) images of mice bearing MCF-7 xenografts and (B) averaged Epf intensity of the bladder ROI, acquired 5 h, 24 h, 48 h or 96 h after exposure to 5NP-1Tz or 5NP-2Tz, labeled with AF660. Epf values have been normalized to the fluorescence intensity of the injected solution in order to keep into account the differences in intrinsic fluorescence emission for each conjugate. The minimum and maximum of the logarithmic colour code in (p/sec/cm^2/sr)/(μW/cm^2) are $1.98 \cdot 10^8$-$8.16 \cdot 10^8$.

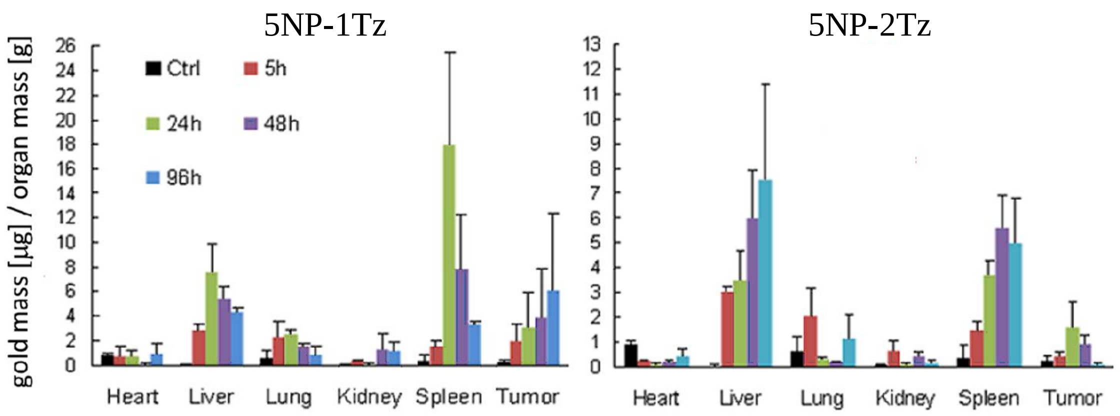

Supplementary Figure 9. Amount of Au accumulated in heart, liver, lung, kidney, spleen and tumor, measured by ICP-MS analysis on organ homogenates. Mean values ± standard deviation of at least 2-3 different animals or samples for each experimental condition. The data for this figure are enlisted in Supplementary Table 6.

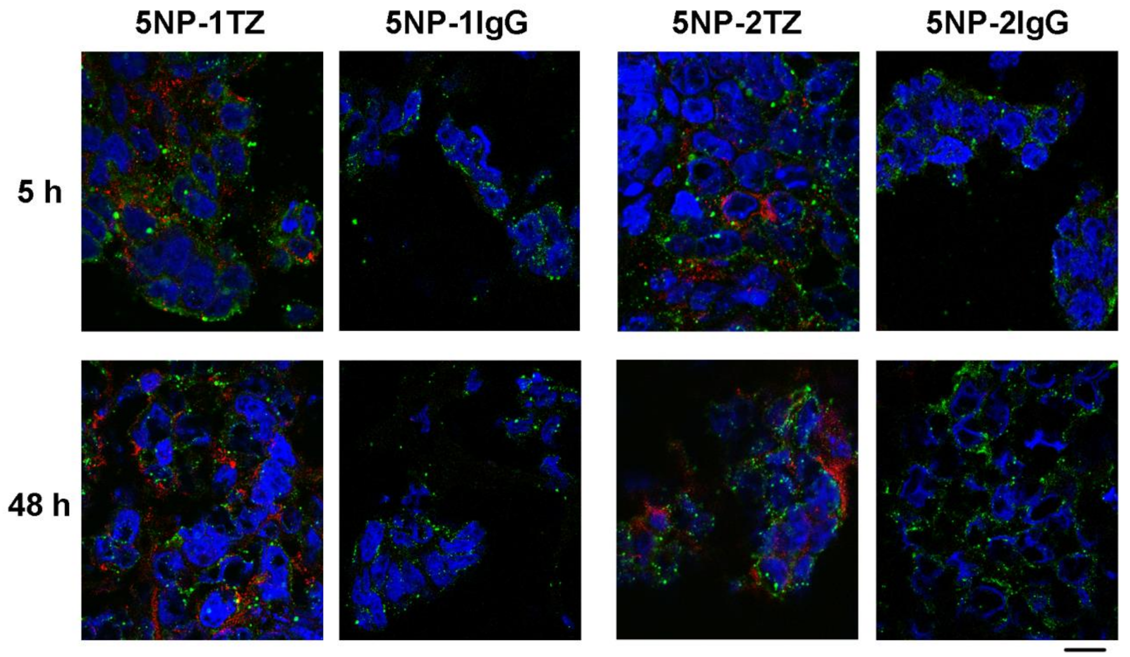

Supplementary Figure 10. Confocal laser scanning micrographs (single optical sections) of cryosections obtained from MCF-7 tumors isolated 5 h or 48 h after exposure to 5NP-1Tz, 5NP-1IgG, 5NP-2Tz, or 5NP-2IgG, labeled with AF660, and then counterstained with anti-cytokeratin19 and DAPI for tumor cells and nuclei detection, respectively. The confocal images of NPs (red) have been overlaid on the corresponding images reporting nuclei (blue) and cells (green). The scale bar corresponds to 10 µm.

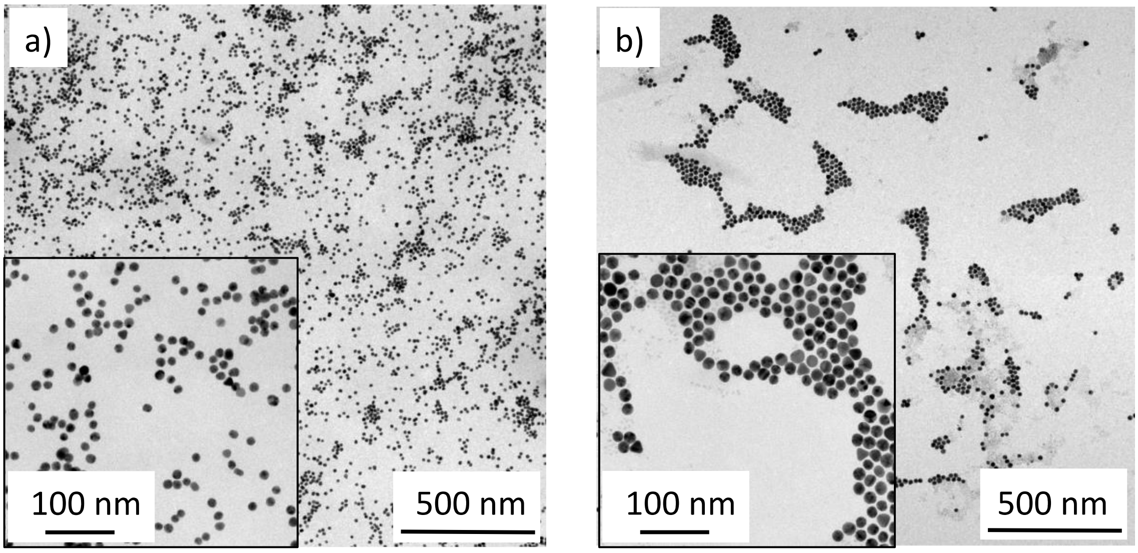

Supplementary Figure 11. TEM images of a) 12NPs and b) 20NPs. The scale bar corresponds to 500 nm and 100 nm in the magnification inset.

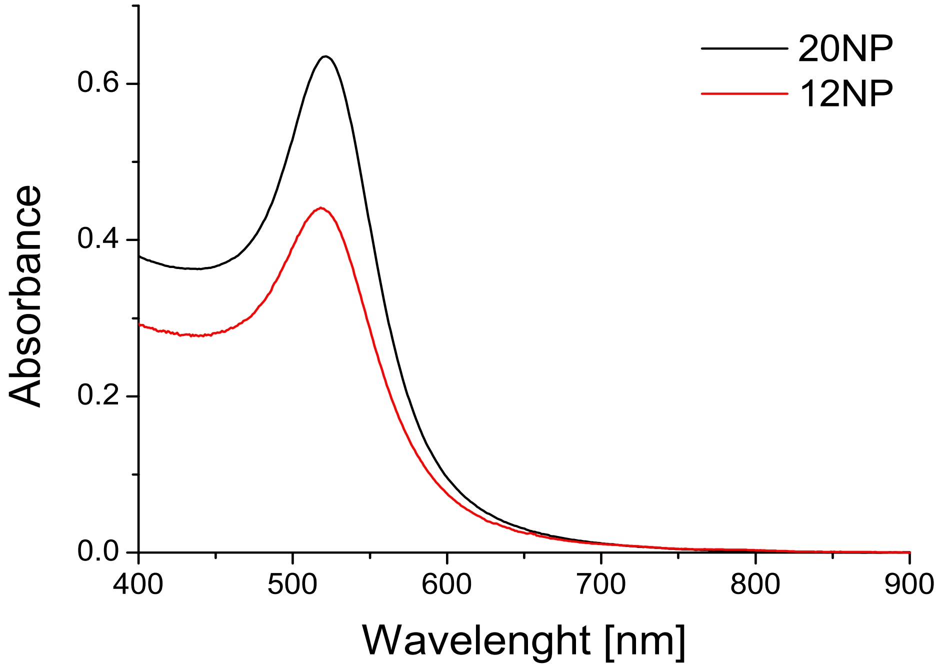

Supplementary Figure 12. Absorption spectrum A(λ) of polymer-coated 12NPs and 20NPs as dissolved in water.

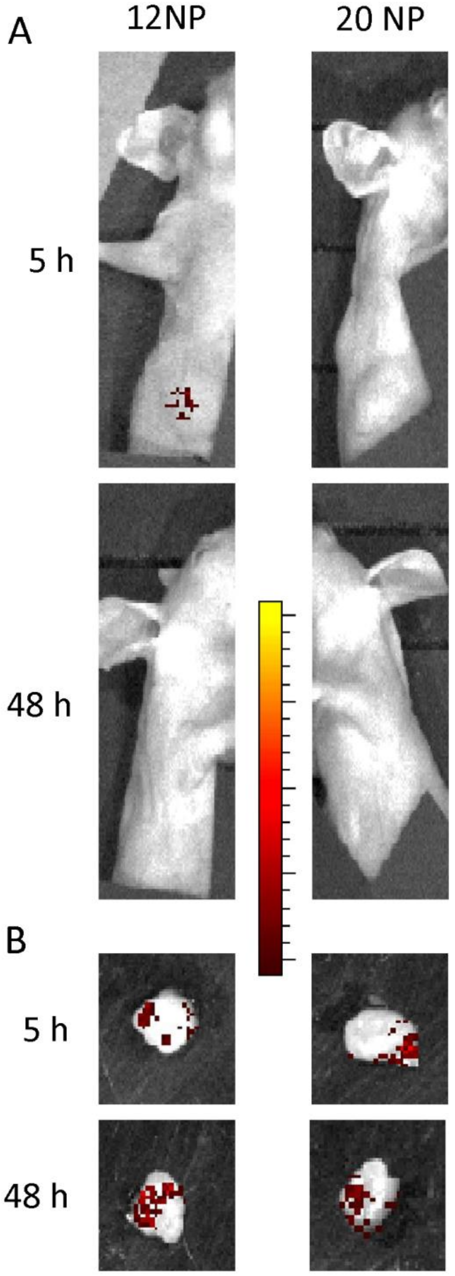

Supplementary Figure 13. (A) Epf images of mice bearing MCF-7 xenografts and (B) isolated tumors, acquired 5 h or 48 h after exposure to 12NP or 20NP labeled with AF660. Epf values have been normalized to the fluorescence intensity of the injected solution in order to keep into account the differences in intrinsic fluorescence emission for each conjugate. The minimum and maximum of the logarithmic colour code in (p/sec/cm^2/sr)/(μW/cm^2) are (from left to right): (A), 5 h: $3.93 \cdot 10^7$-$6.08 \cdot 10^7$, $3.94 \cdot 10^7$-$6.10 \cdot 10^7$; (A) 48 h: $3.91 \cdot 10^7$-$6.07 \cdot 10^7$, $3.91 \cdot 10^7$-$6.08 \cdot 10^7$; (B) 5 h: $1.73 \cdot 10^6$-$1.02 \cdot 10^7$, $1.73 \cdot 10^6$-$1.02 \cdot 10^7$; (B) 48 h: $1.65 \cdot 10^6$-$1.03 \cdot 10^7$, $1.65 \cdot 10^6$-$1.03 \cdot 10^7$

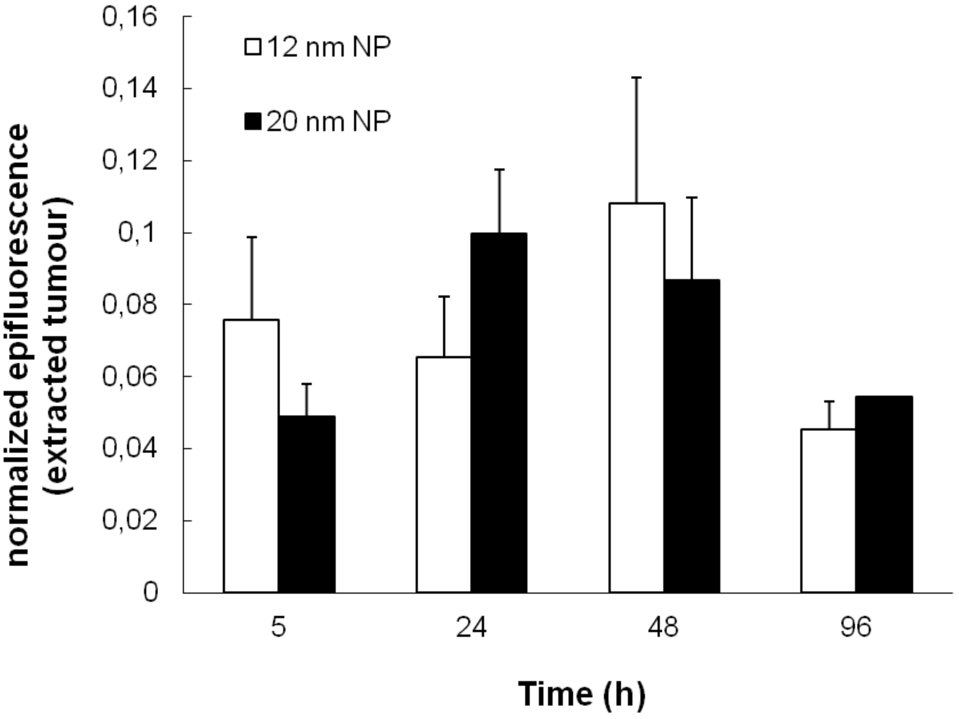

Supplementary Figure 14. Averaged Epf intensity of isolated tumors acquired 5 h, 24 h, 48 h or 96 h after exposure to 12NP or 20NP labeled with AF660. Epf values have been normalized to the fluorescence intensity of injected solution in order to keep into account the differences in intrinsic fluorescence emission for each NP-antibody conjugate. Mean value ± s.e. of 3 different samples for each experimental condition are provided.

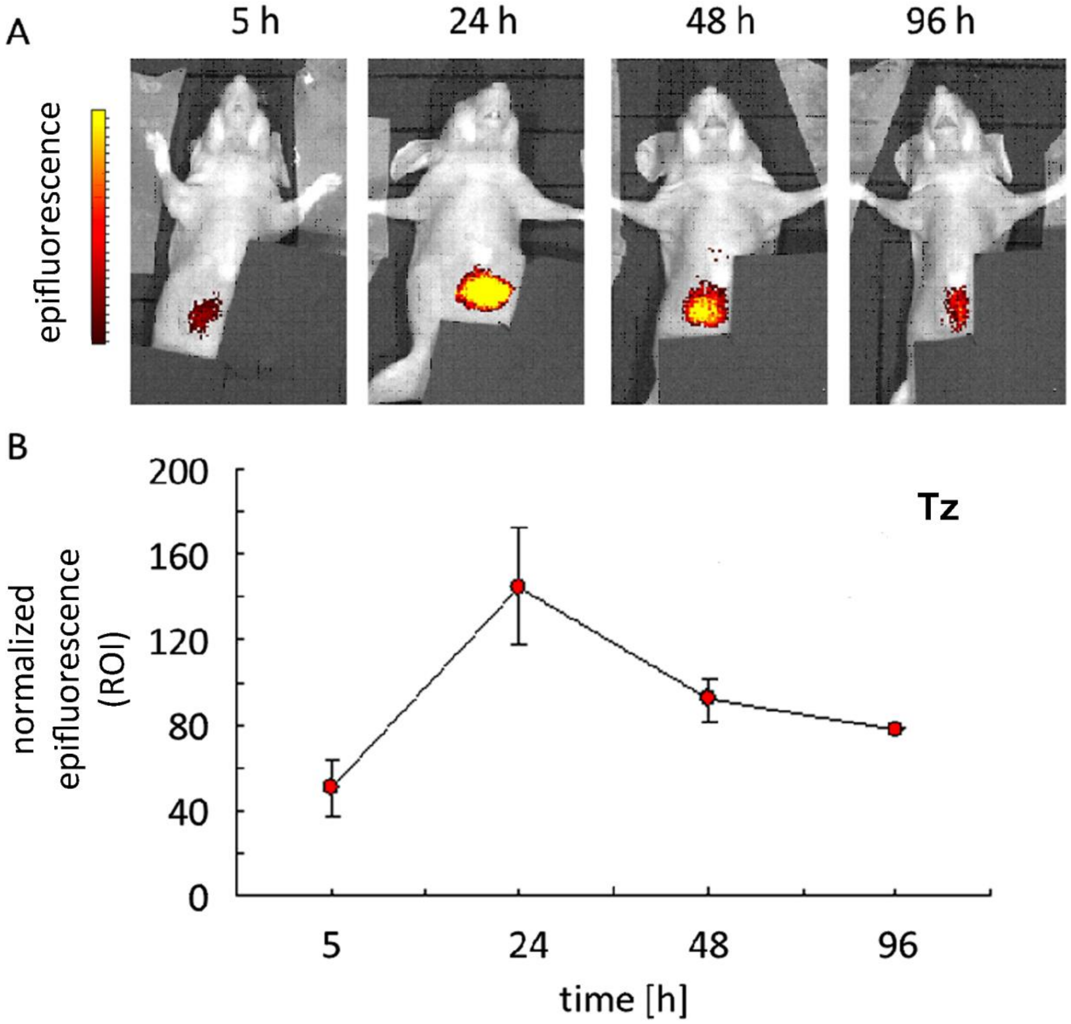

Supplementary Figure 15. Epf images of mice bearing MCF-7 xenografts (minimum and maximum values in colour scale corresponding to $2.99 \cdot 10^7$ and $4.04 \cdot 10^7$(p/sec/cm^2/sr)/(μW/cm^2), where p/sec/cm^2/sr is the number of photons *per* second that leave a square centimeter of tissue and radiate into a solid angle of one steradian (sr), and (B) averaged Epf intensity of the tumor region of interest (ROI), acquired 5 h, 24 h, 48 h or 96 h after exposure to free Tz labeled with AF660 and normalized to the fluorescence intensity of the injected solution. Mean values ± standard error of at least three different animals for each experimental condition are displayed.

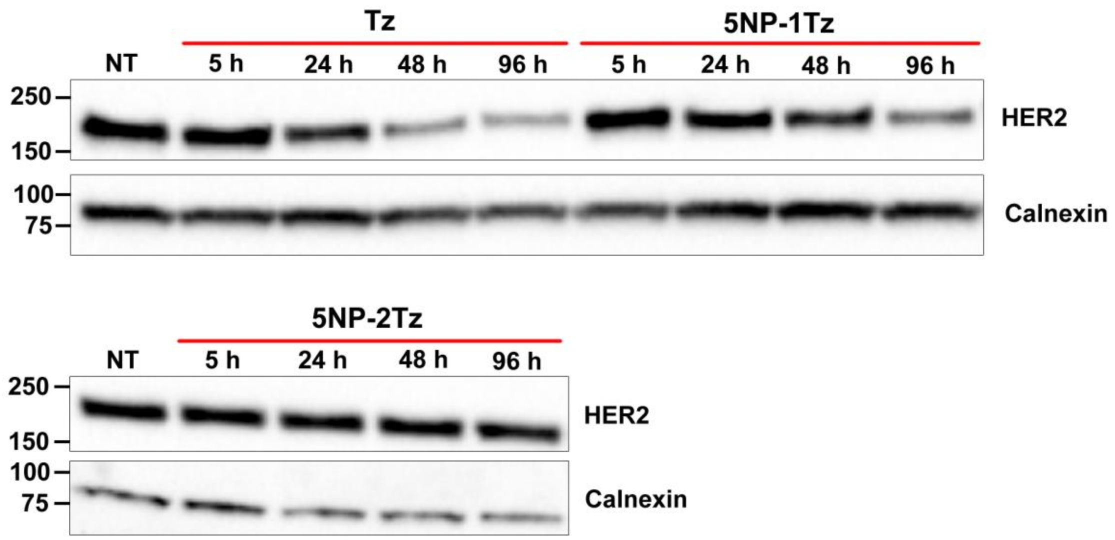

Supplementary Figure 16. HER2 expression in MCF-7 tumor lysates.

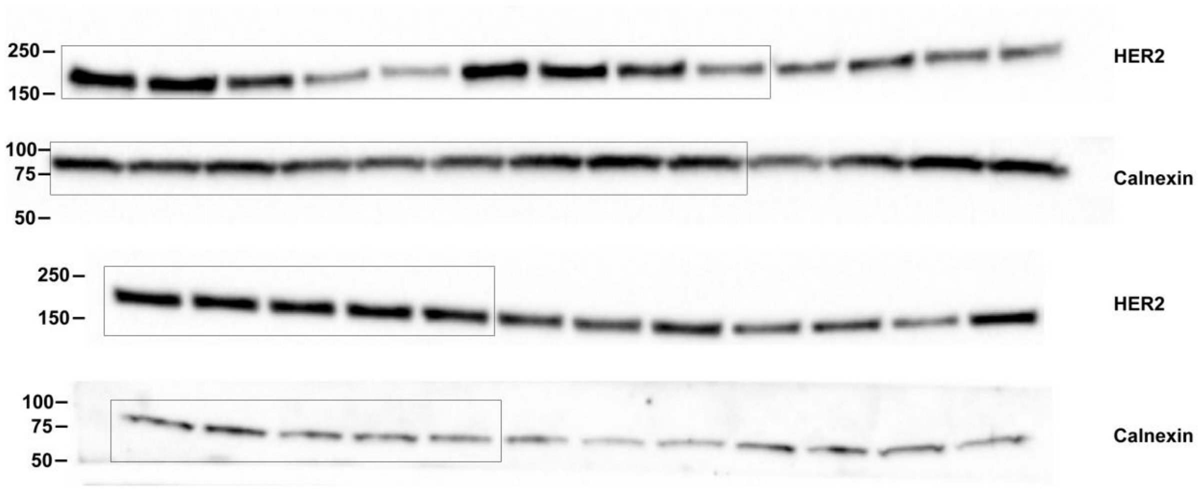

Supplementary Figure 17. Uncropped scans of the immunoblot of Supplementary Figure 16.

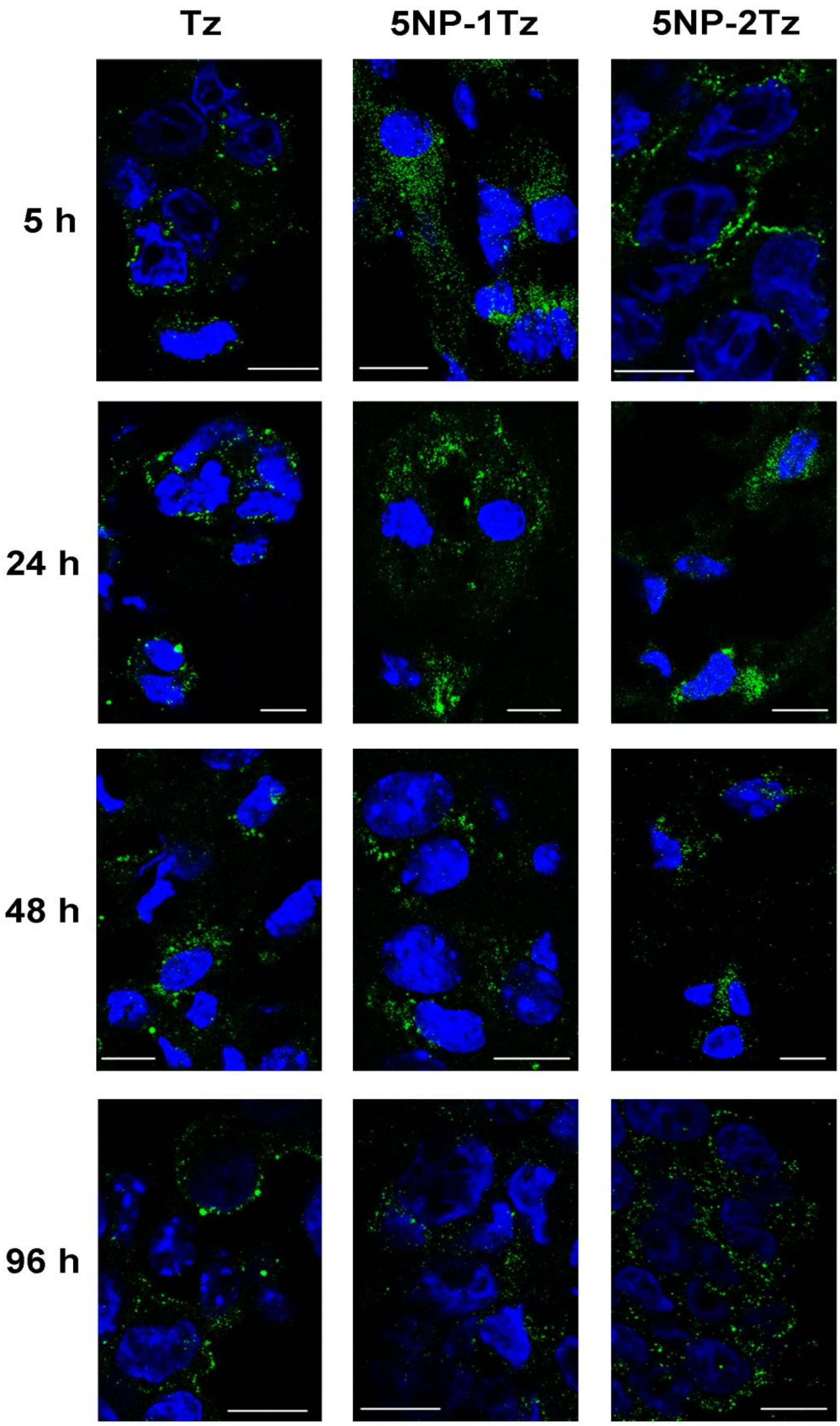

Supplementary Figure 18. Confocal laser scanning micrographs (single optical sections) of cryosections obtained from MCF-7 tumors isolated 5 h, 24 h, 48 h or 96 h after exposure to 5NP-1Tz, 5NP-2Tz, or free Tz, immunodecorated with anti-HER-2 antibody and then counterstained with DAPI for nuclei detection. The confocal images of HER2 expression (green) have been overlaid on the corresponding images reporting nuclei (blue). The scale bars correspond to 10 µm.

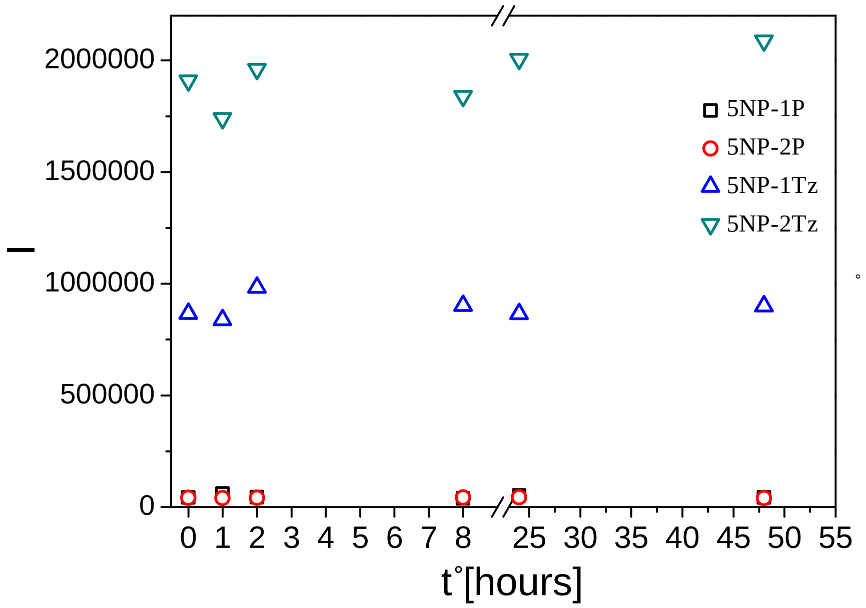

Supplementary Figure 19. Fluorescence quenching experiments of the NPs in human plasma along time.

Supplementary Table 1. Mean hydrodynamic diameter d_h and ζ –potential values of the NPs.

Sample	d_h [nm]	ζ [mV]
5NP	7.7 ± 0.2	-23.7 ± 1.8
5NP-1P	9.6 ± 0.4	-31.5 ± 2.1
5NP-2P	11.1 ± 0.1	-31.6 ± 1.2
5NP-1Tz	11.3 ± 0.4	-34.7 ± 3.3
5NP-2Tz	18.1 ± 0.6	-32.7 ± 0.3

Supplementary Table 2. Mean hydrodynamic diameter d_h

Time (h)	5NP-1Tz (d_h)	5NP-2Tz (d_h)
0	12.3 ± 5.9	18.31 ± 4.7
5	13.4 ± 6.6	17.3 ± 6.5
24	10.6 ± 3.2	22.1 ± 5
48	15.8 ± 5.6	15.9 ± 5.7
96	24.4 ± 7.7	17.45 ± 9.8

Supplementary Table 3. Intensity values at 515 nm upon excitation at 490 nm.

Sample / t [hours]	5NP-1P	5NP-2P	5NP-1Tz	5NP-2Tz
0	42630	41010	865760	$1.91 \cdot 10^6$
1	60580	39840	838310	$1.74 \cdot 10^6$
2	43910	41240	983150	$1.96 \cdot 10^6$
8	38600	41970	901760	$1.84 \cdot 10^6$
24	50940	44390	864750	$2.01 \cdot 10^6$
48	42180	40530	900310	$2.09 \cdot 10^6$

Supplementary Table 4. Normalized intensity values at 515 nm upon excitation at 490 nm. Normalization was done based on the emission collected from the sample at time 0.

t [hours] \ Sample	5NP-1P	5NP-2P	5NP-1Tz	5NP-2Tz
0	1.00	1.00	1.00	1.00
1	1.42	0.97	0.97	0.91
2	1.03	1.01	1.14	1.03
8	0.91	1.02	1.04	0.96
24	1.19	1.08	1.00	1.05
48	0.99	0.99	1.04	1.09

Supplementary Table 5. NP half-life ($t_{1/2}$) values. The mean value is the average of three animals, SD describes the standard deviation.

	$t_{1/2}$ (h)	$t_{1/2}$ (h)	$t_{1/2}$ (h)	Mean $t_{1/2}$ (h)	SD
5NP-1Tz	165.00*	55.44	44.42	49.93	7.79
5NP-2Tz	52.50	44.42	41.01	45.98	5.90
12NP	1.46	0.22	-	0.84	0.87
20NP	13.35	3.29	-	8.32	7.11

Supplementary Table 6. ICP-MS results and calculations for all data sets concerning 5NP-1TZ and 5NP-2TZ NPs injected into mice as well as control sets. m_{Organ} = mass of extracted mouse organ. V_{HNO3} = 2 mL = volume of HNO_3 used for digestion of each organ. α_{dil} = 30 = dilution factor. C'_{Au} = the mass-concentration of elemental gold in diluted solution with dissolved organ as measured with ICP-MS. $\Delta C'_{Au}$ = standard deviation between the three measurements in percent. C_{Au} [ppb] = $C'_{Au} \cdot \alpha_{dil}$ = gold concentration in the original solution with the digested organ. m_{Au} [g] = $C_{Au} \cdot V_{HNO3}$ = total mass of gold in each organ. m_{Au}/m_{Organ} = m_{Au}/m_{Organ}. = mass of gold per mass of organ. As example: (heart 5h 5NP-1TZ): $m_{Au}/m_{Organ} = C'_{Au} * \alpha_{dil} * V_{HNO3} / m_{Organ}$ = 4,07 µg/L * 30 * 0,002 L / 0,1047 g = 2,33 µg/g → $C_{Au}/m_{Organ} = C'_{Au} * \alpha_{dil} / m_{Organ}$ = 4,07 µg/L * 30 / 0,1047 g = 1166,6 ppb/g. Note, that data points far below a measured value of 1 ppb can not be considered as they are below the detection limit of the setup, resulting in very high mean deviation values $\Delta C'_{Au}$.

Type of particle	Time stamp	investi-gated organ	C'_{Au} [ppb]	$\Delta C'_{Au}$ [%]	m_{Organ} [mg]	m_{Au}/m_{Organ} [µg/g]	C_{Au}/m_{Organ} [ppb/g]
5NP-1TZ	5 h	Heart	4.07	4.3305297	0.1047	2.33	1166.687
5NP-1TZ	5 h	Liver	34.93	2.6744479	0.7225	2.90	1450.524

5NP-1TZ	5 h	Lung	0.61	9.2515902	0.1565	0.23	117.3867
5NP-1TZ	5 h	Kidney	1.83	6.9175239	0.2308	0.48	237.6134
5NP-1TZ	5 h	Spleen	3.46	4.7197471	0.0939	2.21	1106.184
5NP-1TZ	5 h	Tumor	0.03	160.57968	0.0507	0.03	15.71194
5NP-1TZ	5 h	Heart	0.02	211.88839	0.1107	0.01	4.61078
5NP-1TZ	5 h	Liver	25.15	3.12395	0.6423	2.35	1174.718
5NP-1TZ	5 h	Lung	14.11	2.8177799	0.1861	4.55	2274.753
5NP-1TZ	5 h	Kidney	1.58	6.5791911	0.287	0.33	165.5876
5NP-1TZ	5 h	Spleen	0.98	7.1690664	0.1077	0.55	273.9693
5NP-1TZ	5 h	Tumor	3.88	6.4910627	0.0498	4.68	2339.341
5NP-1TZ	5 h	Heart	0.12	33.769033	0.0877	0.08	42.30711
5NP-1TZ	5 h	Liver	41.98	5.564306	0.6934	3.63	1816.141
5NP-1TZ	5 h	Lung	5.44	4.6015188	0.1397	2.34	1167.999
5NP-1TZ	5 h	Kidney	15.44	2.9475124	0.2599	3.56	1782.396
5NP-1TZ	5 h	Spleen	2.92	3.7628426	0.094	1.87	932.6401
5NP-1TZ	5 h	Tumor	0.00	N/A	0.0315	0.00	0
5NP-1TZ	24 h	Heart	1.44	4.6294948	0.0922	0.93	466.9288
5NP-1TZ	24 h	Liver	159.57	4.9147313	0.8919	10.73	5367.35
5NP-1TZ	24 h	Lung	7.61	3.8419813	0.1435	3.18	1590.82
5NP-1TZ	24 h	Kidney	1.21	4.5804694	0.2295	0.32	158.6882
5NP-1TZ	24 h	Spleen	34.08	5.1888956	0.0874	23.39	11696.82
5NP-1TZ	24 h	Tumor	0.00	N/A	0.0321	0.00	0
5NP-1TZ	24 h	Heart	1.93	4.3724105	0.0843	1.38	688.193
5NP-1TZ	24 h	Liver	114.80	3.7257469	0.7791	8.84	4420.375
5NP-1TZ	24 h	Lung	3.96	3.7990544	0.1176	2.02	1009.572
5NP-1TZ	24 h	Kidney	0.25	14.689152	0.226	0.07	32.76679
5NP-1TZ	24 h	Spleen	30.10	4.8288318	0.066	27.36	13680.42
5NP-1TZ	24 h	Tumor	2.68	2.8389495	0.0183	8.80	4400.079
5NP-1TZ	24 h	Heart	0.18	19.841051	0.0846	0.13	62.95525
5NP-1TZ	24 h	Liver	48.34	5.5302435	0.9193	3.16	1577.632
5NP-1TZ	24 h	Lung	4.73	4.0283015	0.1085	2.62	1308.784
5NP-1TZ	24 h	Kidney	0.16	20.059226	0.2407	0.04	19.681
5NP-1TZ	24 h	Spleen	3.85	5.7552789	0.0758	3.05	1524.309
5NP-1TZ	24 h	Tumor	0.00	N/A	0.0537	0.00	0
5NP-1TZ	48 h	Heart	0.02	110.58147	0.0672	0.02	9.483834
5NP-1TZ	48 h	Liver	63.42	4.6631524	0.8903	4.27	2137.072
5NP-1TZ	48 h	Lung	1.34	3.6496508	0.1117	0.72	359.9288
5NP-1TZ	48 h	Kidney	0.04	68.973568	0.2478	0.01	4.821925
5NP-1TZ	48 h	Spleen	4.72	3.0129192	0.0727	3.89	1945.947
5NP-1TZ	48 h	Tumor	0.00	N/A	0.0386	0.00	0

5NP-1TZ	48 h	Heart	0.29	13.124427	0.0832	0.21	103.696
5NP-1TZ	48 h	Liver	88.96	4.6480717	0.7396	7.22	3608.311
5NP-1TZ	48 h	Lung	1.23	8.3930832	0.1173	0.63	313.5286
5NP-1TZ	48 h	Kidney	1.49	6.1689716	0.2438	0.37	183.8558
5NP-1TZ	48 h	Spleen	25.00	5.326759	0.0905	16.58	8287.698
5NP-1TZ	48 h	Tumor	0.00	N/A	0.0676	0.00	0
5NP-1TZ	48 h	Heart	0.00	N/A	0.0658	0.00	0
5NP-1TZ	48 h	Liver	46.82	3.6447012	0.555	5.06	2530.934
5NP-1TZ	48 h	Lung	0.83	6.872251	0.1081	0.46	230.258
5NP-1TZ	48 h	Kidney	13.10	5.004387	0.2067	3.80	1900.851
5NP-1TZ	48 h	Spleen	3.84	5.7561351	0.0778	2.96	1480.703
5NP-1TZ	48 h	Tumor	8.54	6.1099131	0.044	11.64	5820.92
5NP-1TZ	96 h	Heart	0.00	N/A	0.0833	0.00	0
5NP-1TZ	96 h	Liver	55.92	4.2967056	0.8378	4.00	2002.393
5NP-1TZ	96 h	Lung	3.43	2.794247	0.1354	1.52	759.1914
5NP-1TZ	96 h	Kidney	0.52	10.306194	0.2361	0.13	65.91879
5NP-1TZ	96 h	Spleen	5.78	4.7839975	0.1075	3.22	1612.211
5NP-1TZ	96 h	Tumor	0.00	N/A	0.0428	0.00	0
5NP-1TZ	96 h	Heart	0.17	12.796655	0.0258	0.39	193.2218
5NP-1TZ	96 h	Liver	67.53	4.5341536	0.8582	4.72	2360.533
5NP-1TZ	96 h	Lung	0.42	8.4035346	0.1256	0.20	99.62371
5NP-1TZ	96 h	Kidney	11.12	4.712753	0.2744	2.43	1215.927
5NP-1TZ	96 h	Spleen	6.83	4.7369409	0.1138	3.60	1800.706
5NP-1TZ	96 h	Heart	3.31	5.3611824	0.0772	2.57	1286.606
5NP-1TZ	96 h	Kidney	4.09	6.6198564	0.2164	1.13	566.7485
5NP-1TZ	96 h	Tumor	6.91	5.7695088	0.0337	12.31	6154.566
5NP-2TZ	5 h	Heart	0.24	6.8875214	0.0846	0.17	84.06365
5NP-2TZ	5 h	Liver	21.92	2.6194818	0.4529<br>9	2.90	1451.934
5NP-2TZ	5 h	Lung	8.16	5.1180026	0.129	3.79	1896.711
5NP-2TZ	5 h	Kidney	1.98	4.9746115	0.1978	0.60	300.8887
5NP-2TZ	5 h	Spleen	2.24	4.9577109	0.0621	2.16	1079.737
5NP-2TZ	5 h	Heart	0.38	10.357847	0.0851	0.27	134.7072
5NP-2TZ	5 h	Liver	0.60	3.866219	0.5945	0.06	30.36827
5NP-2TZ	5 h	Spleen	1.37	6.567642	0.0593	1.38	691.2542
5NP-2TZ	5 h	Kidney	3.39	5.5625106	0.1946	1.05	522.8586
5NP-2TZ	5 h	Tumor	0.30	5.9697767	0.0548	0.33	162.8335
5NP-2TZ	5 h	Heart	0.46	10.511012	0.0863	0.32	161.0682
5NP-2TZ	5 h	Liver	29.09	4.3181459	0.5425	3.22	1608.823

5NP-2TZ	5 h	Lung	4.41	4.5148128	0.113	2.34	1170.89
5NP-2TZ	5 h	Kidney	0.26	13.70748	0.2363	0.07	33.40263
5NP-2TZ	5 h	Spleen	2.09	6.3422979	0.1032	1.21	607.1673
5NP-2TZ	5 h	Tumor	0.49	10.670896	0.0499	0.59	292.7936
5NP-2TZ	24 h	Heart	0.24	8.4102294	0.0749	0.19	96.82937
5NP-2TZ	24 h	Liver	51.81	7.182874	0.5895	5.27	2636.395
5NP-2TZ	24 h	Lung	0.72	9.7611205	0.0993	0.43	216.065
5NP-2TZ	24 h	Kidney	0.46	6.6572624	0.2421	0.11	56.77412
5NP-2TZ	24 h	Spleen	6.93	7.1071418	0.0872	4.77	2383.148
5NP-2TZ	24 h	Tumor	1.66	5.1974179	0.0277	3.59	1794.249
5NP-2TZ	24 h	Heart	0.16	15.256408	0.0756	0.13	65.19976
5NP-2TZ	24 h	Liver	40.36	5.7873924	0.7513	3.22	1611.552
5NP-2TZ	24 h	Lung	0.54	5.9313828	0.1163	0.28	139.0171
5NP-2TZ	24 h	Kidney	0.61	3.912815	0.2173	0.17	84.42526
5NP-2TZ	24 h	Spleen	4.62	4.0607473	0.0822	3.37	1687.33
5NP-2TZ	24 h	Tumor	0.21	10.76576	0.0346	0.36	179.8372
5NP-2TZ	24 h	Heart	0.05	29.925092	0.0759	0.04	21.00945
5NP-2TZ	24 h	Liver	18.84	3.1413947	0.5771	1.96	979.4268
5NP-2TZ	24 h	Lung	0.36	7.6636467	0.1005	0.21	106.4109
5NP-2TZ	24 h	Kidney	0.18	11.245217	0.167	0.07	32.56931
5NP-2TZ	24 h	Spleen	3.24	3.8198165	0.0636	3.05	1526.312
5NP-2TZ	24 h	Tumor	0.47	2.368919	0.0346	0.81	403.9235
5NP-2TZ	48 h	Heart	0.13	9.6202422	0.0718	0.11	54.24814
5NP-2TZ	48 h	Liver	77.16	2.5701997	0.6824	6.78	3392.085
5NP-2TZ	48 h	Lung	0.41	7.9290075	0.1094	0.23	112.5453
5NP-2TZ	48 h	Kidney	5.02	3.4304156	0.2242	1.34	672.3875
5NP-2TZ	48 h	Spleen	4.69	4.3507207	0.0819	3.44	1719.751
5NP-2TZ	48 h	Tumor	0.36	14.768404	0.0279	0.77	382.5808
5NP-2TZ	48 h	Liver	104.97	4.8580451	0.7588	8.30	4150.295
5NP-2TZ	48 h	Lung	0.38	8.8398165	0.1252	0.18	90.34019
5NP-2TZ	48 h	Kidney	2.81	5.0488843	0.2843	0.59	296.9401
5NP-2TZ	48 h	Spleen	7.79	5.6826373	0.086	5.43	2716.096
5NP-2TZ	48 h	Tumor	0.41	5.0110923	0.046	0.54	267.928
5NP-2TZ	48 h	Heart	0.36	12.060914	0.0749	0.29	143.1024
5NP-2TZ	48 h	Liver	36.78	13.41024	0.7379	2.99	1495.473
5NP-2TZ	48 h	Spleen	0.34	14.287168	0.0822	0.25	125.7334
5NP-2TZ	48 h	Tumor	2.17	11.758481	0.0164	7.93	3963.26
5NP-2TZ	48 h	Kidney	6.13	3.0684597	0.2305	1.60	797.6222
5NP-2TZ	96 h	Heart	0.14	21.888283	0.0862	0.10	47.8598
5NP-2TZ	96 h	Liver	48.46	5.2083371	0.6135	4.74	2369.631

5NP-2TZ	96 h	Lung	0.46	14.998616	0.12	0.23	114.6115
5NP-2TZ	96 h	Kidney	0.20	9.8301402	0.2392	0.05	24.8021
5NP-2TZ	96 h	Spleen	4.57	2.4657763	0.0882	3.11	1555.058
5NP-2TZ	96 h	Tumor	0.00	N/A	0.007	0.00	0
5NP-2TZ	96 h	Heart	0.22	26.594451	0.0838	0.15	77.29624
5NP-2TZ	96 h	Liver	50.83	10.620399	0.7572	4.03	2013.774
5NP-2TZ	96 h	Kidney	0.87	9.7041908	0.2558	0.20	101.9894
5NP-2TZ	96 h	Spleen	5.39	0.5472526	0.0988	3.27	1637.475
5NP-2TZ	96 h	Tumor	0.01	70.495511	0.0333	0.03	13.40985
5NP-2TZ	96 h	Heart	1.15	5.194264	0.0683	1.01	505.1771
5NP-2TZ	96 h	Liver	127.63	10.021561	0.5528	13.85	6926.259
5NP-2TZ	96 h	Lung	3.62	10.555634	0.1019	2.13	1065.72
5NP-2TZ	96 h	Kidney	0.96	4.5904229	0.1999	0.29	143.9158
5NP-2TZ	96 h	Spleen	13.30	2.2127304	0.093	8.58	4289.244
5NP-2TZ	96 h	Tumor	0.05	8.7400118	0.0127	0.22	112.2322
Ctrl 1		Heart	1.03	3.5905844	0.0852	0.73	362.856
Ctrl 1		Liver	1.63	5.5346087	0.717	0.14	68.38426
Ctrl 1		Kidney	0.45	7.6984927	0.2064	0.13	64.96589
Ctrl 1		Spleen	0.49	5.6576273	0.0581	0.51	254.8914
Ctrl 1		Tumor	0.01	7.448756	0.0246	0.001	2.115854
Ctrl 2		Heart	1.15	8.571556	0.0755	0.91	455.5448
Ctrl 2		Liver	0.80	4.3489226	0.6717	0.07	35.52866
Ctrl 2		Lung	1.93	4.4807696	0.094	1.23	616.3588
Ctrl 2		Kidney	0.43	9.5525656	0.205	0.13	62.62923
Ctrl 2		Spleen	0.60	8.8574378	0.0541	0.66	331.1462
Ctrl 2		Tumor	0.45	6.4446952	0.0324	0.84	418.6209
Ctrl 3		Heart	1.40	5.0752095	0.0718	1.17	583.1921
Ctrl 3		Liver	0.11	4.2446455	0.6518	0.01	5.248946
Ctrl 3		Lung	0.07	11.142071	0.0897	0.05	25.03155
Ctrl 3		Kidney	0.09	13.818674	0.1883	0.03	13.94967
Ctrl 3		Spleen	0.00	131.90421	0.0515	0.01	2.534146
